# Biliary atresia-related liver fibrosis

**DOI:** 10.3389/fcell.2026.1905495

**Published:** 2026-08-11

**Authors:** Tao Zhou, Fang Wang, Pengpeng Sun, Meng Kong

**Affiliations:** 1 Department of Pediatric Surgery, Jinan Maternity and Child Care Hospital Affiliated to Shandong First Medical University, Jinan, China; 2 Department of Hepatobiliary Surgery, Children’s Hospital of Chongqing Medical University, National Clinical Research Center for Child Health and Disorders, Ministry of Education Key Laboratory of Child Development and Disorders, Chongqing Key Laboratory of Child Rare Diseases in Infection and Immunity, Chongqing Key Laboratory of Structural Birth Defect and Reconstruction, Chongqing, China; 3 Department of Pediatric Surgery, Dazhou Central Hospital, Dazhou, China; 4 Department of Nutrition, Dazhou Central Hospital, Dazhou, China; 5 Department of Medical Laboratory, Jining No.1 People’s Hospital, Jining, China; 6 Department of Pediatric Surgery, Children’s Hospital Affiliated to Shandong University, Jinan, China; 7 Department of Pediatric Surgery, Jinan Children’s Hospital, Jinan, China

**Keywords:** biliary atresia, hepatic stellate cell, liver fibrosis, matrix metalloproteinase-7, shear wave elastography

## Abstract

Biliary atresia (BA) is a devastating cholestatic disease of infancy characterized by progressive fibro-obliterative cholangiopathy, which, without timely intervention, inexorably leads to end-stage liver disease and the need for liver transplantation. Hepatic fibrosis constitutes not only the histopathological cornerstone of BA but also the principal determinant of disease trajectory, native liver survival, and long-term outcomes. This review provides a comprehensive synthesis of the current understanding and unmet clinical needs in BA-associated liver fibrosis, systematically addressing three interconnected domains: pathogenesis, diagnostic modalities, and therapeutic strategies. In the pathogenic realm, we dissect the multilayered molecular architecture-from genetic susceptibility loci (including ciliopathy-related PKD1L1, developmental regulators ADD3,GPC1, and immune-modulating polymorphisms) through aberrant cellular activation (biliary epithelial cells undergoing ductular reaction and epithelial-mesenchymal transition, hepatic stellate cells and portal fibroblasts transdifferentiating into myofibroblasts, and immune cells polarizing toward pro-fibrotic phenotypes), to the integrated signaling network of TGF-β/Smad, Wnt/β-catenin, Hippo/YAP, and Notch pathways, further sustained by epigenetic modifications involving DNA methylation, histone remodeling, and non-coding RNAs. In diagnostics, we evaluate conventional ultrasound, emerging shear wave elastography, serum matrix metalloproteinase-7 as a promising biomarker, and risk-stratification algorithms, while critically comparing their strengths and limitations. In therapeutics, we assess the enduring role of Kasai portoenterostomy (KPE), the limitations of current adjunctive therapies, and the evolving landscape of pharmacological interventions-including ileal bile acid transporter inhibitors (odevixibat), farnesoid X receptor agonists (obeticholic acid), eicosapentaenoic acid, and N-acetylcysteine-alongside ongoing clinical trials. We further highlight how single-cell and spatial transcriptomic technologies are revolutionizing our comprehension of cellular heterogeneity and intercellular crosstalk within the fibrotic niche, offering unprecedented opportunities for molecular subtyping and precision medicine. By bridging mechanistic insights with clinical translation, this review provides a conceptual framework to guide future research, improve diagnostic precision, and develop rationally targeted combination therapies for BA-associated liver fibrosis.

## 1 Introduction

Biliary atresia (BA) is the most common progressive obstructive disease of the bile ducts in neonates and is primarily characterized by fibrosis of the intrahepatic and extrahepatic bile ducts and bile buildup1. Without timely intervention, patients typically progress to end-stage liver disease within a few years, ultimately requiring liver transplantation (Kong and Xiang, 2021). Many literature report a close relationship between BA and liver fibrosis, where liver fibrosis is not only one of the pathological manifestations of BA but also an important factor affecting patient prognosis and the need for liver transplantation (Wu et al., 2022). Therefore, understanding the pathogenesis, diagnostic advances, and treatment strategies of liver fibrosis related to BA is crucial for improving the quality of life of patients.

Currently, the pathogenesis of liver fibrosis related to BA is not fully understood. Many studies suggest that the onset of BA may be associated with various factors, including genetic factors, environmental factors, and immune responses (Chen et al., 2025). Some studies have reported specific genetic variations in BA patients, such as copy number variations and single nucleotide polymorphisms, which may be related to the pathogenesis of BA (Tam et al., 2024). Additionally, inflammatory responses and changes in the extracellular matrix are important factors driving the progression of liver fibrosis. Research indicates that in the context of BA, liver fibrosis is closely linked to bile buildup, liver cell damage, and a reduced ability to regenerate (Cavallo et al., 2022). In terms of diagnosis, early identification of BA is crucial. Currently, imaging examinations, such as ultrasound, have become the preferred tool for diagnosing BA. In recent years, emerging ultrasound SWE technology has been studied for assessing the degree of liver fibrosis, showing high accuracy in distinguishing BA from other causes of neonatal jaundice (Sandberg et al., 2021).

Furthermore, researchers have proposed a risk stratification-based diagnostic algorithm that improves the diagnostic accuracy of BA by analysing ultrasound results and biochemical indicators (Dong et al., 2022). Despite progress in understanding the pathogenesis and diagnostic methods of BA, effective antifibrotic treatment methods are lacking. Traditionally, BA treatment relies primarily on surgical intervention to reconstruct the bile ducts. However, the improvement of liver fibrosis after surgery remains a significant challenge (Fligor et al., 2022). Therefore, recent studies have explored new pharmacological treatment strategies, including anti-inflammatory drugs, antifibrotic agents, and stem cell therapy, in hopes of improving the prognosis of BA patients and delaying the progression of liver fibrosis (Lee and Seki, 2023). In summary, research on liver fibrosis related to BA is ongoing, and future studies need to further clarify its pathogenesis, optimize diagnostic methods, and explore more effective treatment strategies to improve patient survival rates and quality of life. This review will comprehensively describe the pathological mechanisms, diagnostic methods, and therapeutic strategies of BA-associated fibrosis, thereby providing enhanced insights into the treatment of BA.

## 2 Pathogenesis of liver fibrosis related to BA

### 2.1 Genetic factors and BA-related genes

As the core pathological endpoint of BA, liver fibrosis arises not from a monogenic defect but from a complex interplay between genetic susceptibility, environmental triggers, and molecular signaling cascades. Rather than operating in isolation, these genetic factors collectively converge upon a shared pathogenic axis that drives progressive fibrogenesis: primary cholangiocyte injury/dysplasia, sustained immune-mediated inflammation, hepatic stellate cell (HSC) activation, and excessive extracellular matrix (ECM) deposition. Based on current evidence, we can stratify these susceptibility genes into distinct functional clusters, and critically, integrate findings from human cohort studies with mechanistic insights from animal models to clarify their hierarchical importance.

Using modern genome and exome sequencing approaches, multiple research groups worldwide have identified contributions of ciliary and other gene variants to the pathogenesis of BA (Lam et al., 2021; Rajagopalan et al., 2020; Tran et al., 2021). Ciliopathy-driven developmental defects as a primary trigger. Among all genetic contributors, ciliopathy-related variants-particularly in PKD1L1-currently possess the strongest translational evidence linking human BA with animal phenotypes. PKD1L1 encodes a polycystin-family protein localized to primary cilia and the plasma membrane, where it regulates calcium signaling and mechanotransduction during embryonic organogenesis (Berauer et al., 2019). Multicenter genetic explorations of BA with splenic malformation initially identified PKD1L1 mutations as candidate etiologic contributors (Berauer et al., 2019). Critically, subsequent animal studies have provided direct mechanistic validation: liver-restricted PKD1L1 deletion in mice recapitulates key features of human BA, including delayed biliary maturation, cholangiocyte hyperproliferation, peribiliary fibrogenic inflammation, and arterial hypertrophy (Fabris et al., 2019; Frassetto et al., 2018). Bile duct ligation further exacerbates these lesions in PKD1L1-deficient mice, confirming a primed susceptibility to fibrotic stress, whereas wild-type controls remain resistant (Fabris et al., 2019; Frassetto et al., 2018). These findings firmly establish a ciliopathic etiology in a subset of BA, and the PKD1L1-deficient model offers a valuable platform for testing novel therapeutic strategies (Hellen et al., 2023; Lim et al., 2024). Beyond PKD1L1, ciliary and left-right patterning genes such as KIF3B, CFC1, ZIC3, INV, and TTC17 also contribute to abnormal embryonic visceral and vascular patterning in syndromic BA, thereby exacerbating early fibrotic progression (Schön et al., 2002; So et al., 2020).

Developmental defects and cholangiocyte differentiation arrest. During embryonic biliary development, the NOTCH, TGF-β, BMP, FGF, and Hedgehog signaling pathways precisely regulate ductal plate remodeling (Lin et al., 2018; Shaalan et al., 2019; Meng et al., 2025). Importantly, variants in ADD3 (specifically rs17095355) and GPC1 (copy number deletions and rs2292832 polymorphisms) are not merely animal-derived candidates but have been robustly replicated as susceptibility loci across multiple independent human populations (Garcia-Barceló et al., 2010; Zeng et al., 2014; Ke et al., 2016). These genes, along with SOX9, HNF1β, JAG1, and CD56, are mechanistically linked to ductal plate malformation and arrested cholangiocyte differentiation (Bai et al., 2020; Lisboa et al., 2011; Naydenov and Ivanov, 2010; Zhang et al., 2016). By disturbing the spatiotemporal regulation of bile duct formation, they establish an innate structural vulnerability that predisposes to subsequent fibrotic insult.

Metabolic barrier disruption and immune dysregulation amplify injury. The transition from developmental vulnerability to progressive fibrosis relies heavily on postnatal triggers involving bile acid toxicity and leaky barrier function. Human polymorphisms in ABCB11 impair the bile salt export pump, promoting intrahepatic bile acid accumulation and cholangiotoxic injury (Van Tung et al., 2021). Concurrently, CDC42 deficiency disrupts hepatocyte tight junctions (Zhou et al., 2021), and reduced EFEMP1 expression compromises the structural integrity of elastic fibers in the bile duct (Gupta et al., 2025). Collectively, these genetic insults compromise ductal barrier integrity, triggering bile leakage and persistent peribiliary inflammation. At the immune level, epigenetic abnormalities-specifically IFN-γ hypomethylation and FOXP3 promoter hypermethylation-impair regulatory T-cell function and tilt the Th1/Th2 balance toward a pro-inflammatory state, effectively amplifying the fibrotic cascade (Mack et al., 2004; Liu et al., 2019; Li K. et al., 2016).

Pro-fibrotic gene polymorphisms drive HSC activation and ECM remodeling. In the final effector phase, polymorphisms and dysregulated expression of canonical fibrotic drivers mediate HSC activation and ECM deposition. TGF-β, CTGF variants enhance epithelial-mesenchymal transition (EMT) and collagen synthesis (Kurabekova et al., 2023; Li F. B. et al., 2016); the rs9690350 polymorphism in PDGFA, combined with DNA hypomethylation, potently stimulates HSC proliferation (Liu et al., 2020; Cofer et al., 2016). Furthermore, VEGFA dysregulation induces intrahepatic vascular malformation and tissue hypoxia, establishing a self-perpetuating fibrosis-hypoxia-vascular abnormality cycle (Allam et al., 2019; Edom et al., 2011). Compounding this, elevated MMP7/MMP2 alongside TIMP1 upregulation dysregulates matrix metalloproteinase balance, while abnormal hyaluronic acid deposition mechanically compresses periductal regions, accelerating biliary obliteration (Nadler et al., 2009). Finally, non-coding RNAs such as miR-29c and miR-200b amplify these fibrotic signals by post-transcriptionally targeting key matrix remodeling pathways (Wang et al., 2019; Xiao et al., 2015).

**FIGURE 1:**
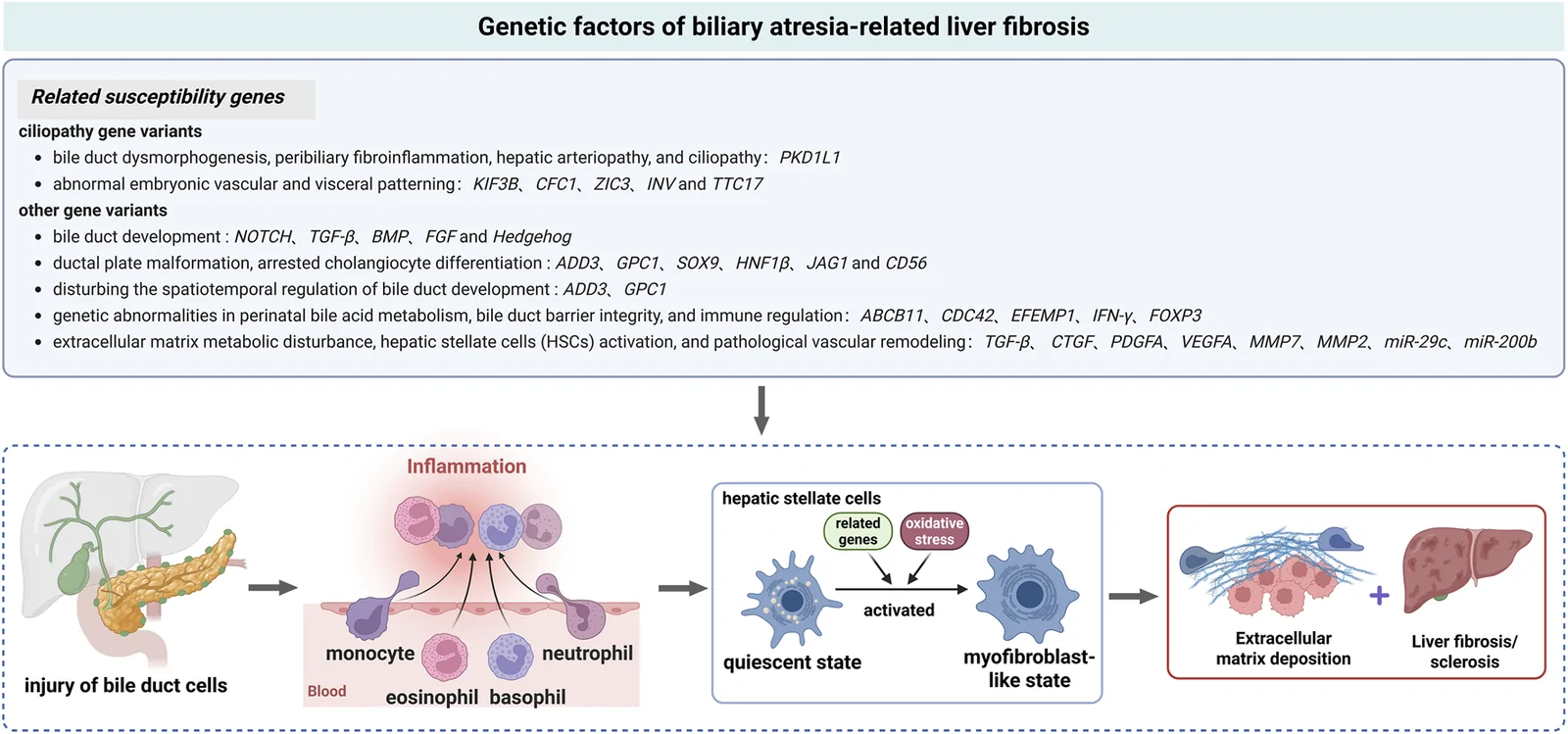
Genetic susceptibility and molecular mechanisms driving liver fibrosis in BA. There are two types of genetic susceptibility factors: cilia disorder gene mutations (e.g., PKD1L1, KIF3B, CFC1) disrupt bile duct morphogenesis and embryonic vascular patterning, while other gene mutations (e.g., NOTCH, TGF-β, ADD3, GPC1, SOX9) affect bile duct plate formation, bile duct cell differentiation, and their timely regulation; Concurrently, genes such as ABCB11, CDC42, and FOXP3 are involved in perinatal bile acid metabolism, barrier integrity, and immune regulation, while TGF-β, MMP7, and miR-29c trigger ECM metabolic dysregulation. These genetic defects collectively lead to bile duct cell dysfunction, which in turn recruits inflammatory cells such as monocytes and neutrophils to infiltrate the tissue and release pro-inflammatory factors. Under sustained inflammatory stimulation, quiescent hepatic stellate cells are activated and transdifferentiate into a myofibroblast-like phenotype, leading to massive deposition of ECM components such as collagen. Concurrently, factors such as TGF-β, CTGF, and VEGFA drive pathological vascular remodeling, while imbalances in the regulation of MMPs and miRNAs further exacerbate ECM accumulation. Ultimately, these mechanisms synergistically drive the progression of liver fibrosis and cirrhosis.

In summary, the genetic landscape of BA-related liver fibrosis is not a random list of isolated genes but a functionally hierarchical network. While animal models (e.g., PKD1L1-KO) provide critical mechanistic proof-of-concept, human replication studies (particularly for ADD3 and GPC1) offer the strongest population-level evidence for clinical susceptibility. These distinct genetic tiers-spanning developmental defects, metabolic barrier failure, immune dysregulation, and pro-fibrotic signaling-ultimately converge on a final common pathway: cholangiocyte injury-peribiliary inflammation-HSC activation-ECM overproduction. This unified conceptual framework highlights potential stage-specific therapeutic targets and underscores the need for multi-omics integration in future BA research (Figure 1).

### 2.2 Abnormally activated cells contributed to BA fibrosis

Liver fibrosis is a critical link in the progression of BA, whose essence is a chronic pathological process characterized by abnormal activation and interaction of various intrahepatic cells, leading to excessive deposition of ECM and remodeling of tissue structure. In the complex pathological process of BA-associated liver fibrosis, it is not a single cell but a variety of cell types that are involved in abnormal activation and dysfunction. These cells cooperate with each other to form a complex regulatory network, which collectively promotes the occurrence and development of fibrosis. Among them, the most critical abnormally activated cells include bile duct epithelial cells (BECs) (Li et al., 2026), HSCs (Ramm et al., 1998), immune cells (Li et al., 2026), and portal fibroblasts (PFs) (Zagory et al., 2015). There are significant differences in the activation phenotypes of different cells, and their molecular mechanisms involved in fibrosis also have their own focuses. The following section will elaborate on the biological characteristics, activation mechanisms of these cells, as well as how they participate in the pathological process of BA-associated liver fibrosis through direct or indirect effects, so as to provide theoretical support for in-depth understanding of the pathogenesis of BA-associated liver fibrosis and the search for potential targets for targeted intervention.

#### 2.2.1 Bile duct epithelial cells (BECs)

As the primary target cells of liver injury in BA, BECs are not only direct victims of pathological damage but also core initiators and key drivers of liver fibrosis progression (Ayabe et al., 2025). Under physiological conditions, BECs are in a quiescent state, maintaining the structural integrity of bile ducts and the normal function of bile excretion (Park, 2012). However, under the stimulation of various pathogenic factors such as perinatal viral infection, immune disorders, bile acid accumulation, and genetic susceptibility, BECs undergo abnormal activation, triggering a series of phenotypic changes and functional disorders (Ortiz-Perez et al., 2020; Vij and Rela, 2020; Mack, 2007). Through autocrine/paracrine effects and interactions with other intrahepatic cells, BECs participate in the occurrence and development of BA-associated fibrosis, and together with HSCs, immune cells, and PFs, form a complex regulatory network (Fabris et al., 2017).

The abnormal activation of BECs in BA is first manifested as ductular reaction (DR), which refers to the abnormal proliferation of BECs and the formation of a large number of immature duct-like structures, and this is also one of the characteristic histological changes of BA (Priester et al., 2010). Under normal circumstances, BECs have weak proliferative capacity; however, under injury stimulation, their proliferative activity is significantly enhanced, and a large number of proliferative duct-like structures appear in the portal area, accompanied by the loss of cell polarity and disruption of tight junctions (Zhou et al., 2021; Lou et al., 2025). This further leads to stenosis, obstruction, or dilation of the bile duct lumen, aggravating bile stasis and forming a vicious cycle of bile stasis-bile duct injury-further stasis (Lui, 2025). The severity of DR is closely related to the grade of BA-associated liver fibrosis and the native liver survival rate of children (Sutton et al., 2024). Even if bile drainage is successfully restored through KPE, the abnormal activation of BECs and DR may still persist, which is also an important reason for the continuous progression of postoperative liver fibrosis and the need for liver transplantation in some children eventually (Sutton et al., 2024; Xiao et al., 2024).

EMT is the core phenotype of abnormal BECs activation and a key mechanism by which BECs participate in fibrosis. Quiescent BECs express the epithelial cell marker E-cadherin, which maintains the morphology and function of epithelial cells. After activation, BECs gradually lose their epithelial phenotype and acquire mesenchymal cell characteristics, which is manifested by downregulated expression of E-cadherin and upregulated expression of mesenchymal markers such as N-cadherin, Vimentin, and α-smooth muscle actin (α-SMA) (Siyu et al., 2022). BECs that undergo EMT can directly synthesize and secrete ECM components such as type I/III collagen and fibronectin, and simultaneously secrete pro-fibrotic factors, directly promoting the progression of liver fibrosis (Wells, 2008; Omenetti and Diehl, 2011). Studies have confirmed that the Hedgehog signaling pathway is significantly activated in BA liver tissues, which can induce EMT of BECs by regulating transcription factors such as Snail and Twist, and is an important molecular mechanism for BECs activation in BA (Omenetti et al., 2011a).

Activated BECs also release a variety of pro-inflammatory and pro-fibrotic factors through autocrine/paracrine pathways, construct a pro-fibrotic microenvironment, and indirectly regulate the progression of liver fibrosis. Among them, TGF-β, platelet-derived growth factor (PDGF), and connective tissue growth factor (CTGF) are core pro-fibrotic factors, which can directly affect HSCs and PFs, promote their transformation into myofibroblasts, and enhance ECM synthesis capacity (Fabris et al., 2017). Meanwhile, activated BECs can also secrete pro-inflammatory factors such as interleukin-6 (IL-6) and tumor necrosis factor-α (TNF-α), recruit immune cells such as macrophages and T lymphocytes to infiltrate liver tissues, amplify the inflammatory response, and further aggravate bile duct injury and fibrosis (Udomsinprasert et al., 2022). In addition, the Hippo signaling pathway effector YAP1 is highly expressed in BA BECs. After binding to the co-activator EP300, YAP1 can directly regulate the transcription of the downstream target gene plasminogen activator inhibitor-1 (SERPINE1), inhibit ECM degradation, and accelerate the progression of fibrosis (Zhu et al., 2026).

The amplification effect of bile acid toxicity is also closely related to BECs activation. BECs injury leads to impaired bile excretion, resulting in massive intrahepatic accumulation of bile acids (such as cholic acid and deoxycholic acid) (Fligor et al., 2022). The accumulated bile acids can further induce BECs activation, proliferation, and EMT by activating bile acid receptors on the surface of BECs (such as G protein-coupled bile acid receptor TGR5), and simultaneously promote the secretion of pro-inflammatory and pro-fibrotic factors, forming a vicious cycle of bile stasis-BEC activation- inflammation-fibrosis and accelerating the progression of BA-associated liver fibrosis (Park, 2012; Zeng J. et al., 2023).

#### 2.2.2 Hepatic stellate cells (HSCs)

**FIGURE 2:**
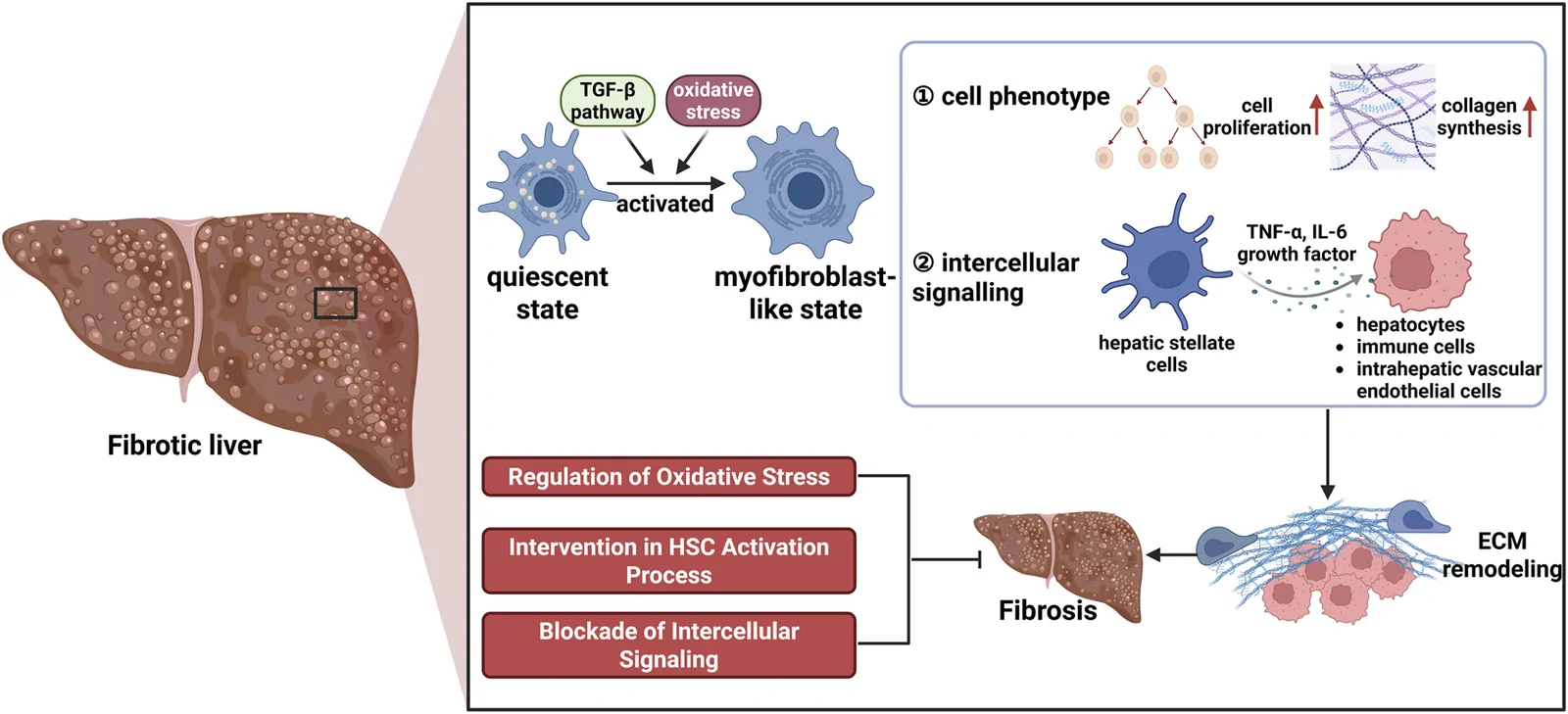
The role of HSCs in the mechanism of BA-related liver fibrosis. The mechanism by which HSCs are activated includes stimulation by liver injury/pathological conditions, activation through the TGF-β signalling pathway (main activating factor), the oxidative stress response, and other triggering factors; after activation, phenotypic changes occur, resulting in differentiation from a quiescent state to a myofibroblast-like state, with increased proliferation and collagen synthesis capacity. In terms of intercellular signalling, HSCs can secrete inflammatory factors (such as TNF-α and IL-6) and growth factors and transmit signals through an interactive network with hepatocytes, intrahepatic vascular endothelial cells, and immune cells. Its pathological effects are reflected in its participation in extracellular matrix remodelling (increased collagen secretion, changes in other matrix components, destruction of the liver microenvironment and structure) and promotion of fibrosis (increasing the degree of cirrhosis and accelerating the process of fibrosis). On the basis of these findings, potential therapeutic targets can focus on interventions in the HSC activation process, blocking intercellular signalling, and regulating oxidative stress, providing a theoretical direction for the treatment of BA-related liver fibrosis.

In the pathological microenvironment of BA, quiescent HSCs are abnormally activated and gradually transform into myofibroblasts (MFs) under the stimulation of various injury signals, representing a key node in the progression of BA-associated liver fibrosis (Kitto and Henderson, 2021) (Figure 2). The activation of HSCs in BA is not caused by a single factor but results from the joint regulation of multiple signals, with core inducing factors originating from pro-fibrotic factors secreted by BECs and immune cells, as well as the toxic effect of accumulated bile acids (Fabris et al., 2017). TGF-β, mainly secreted by activated BECs and macrophages, is the most potent factor for activating HSCs (Fabris et al., 2017). By binding to specific receptors on HSC surfaces, TGF-β activates the Smad2/3 signaling pathway, directly upregulating fibrosis-related genes such as collagen type I/III, fibronectin, and CTGF, and promoting HSC-to-MF transformation (Kitto and Henderson, 2021). PDGF, primarily secreted by activated BECs, platelets, and macrophages-particularly the PDGF-BB subtype-enhances HSC proliferation and migration through the PI3K/AKT signaling pathway, promoting their aggregation to liver injury sites and amplifying the fibrotic effect (Kitto and Henderson, 2021). Pro-inflammatory factors such as IL-1β and TNF-α, secreted by infiltrating immune cells in BA liver tissues, activate NF-κB and MAPK signaling pathways and act synergistically with TGF-β to accelerate HSC activation (Udomsinprasert et al., 2022). Additionally, bile acid accumulation caused by impaired bile excretion in BA directly induces HSC activation by activating bile acid receptors (such as TGR5) on HSC surfaces, while simultaneously promoting their secretion of pro-fibrotic factors, forming a positive cycle of bile stasis-HSC activation-fibrosis (Kitto and Henderson, 2021).

**FIGURE 3:**
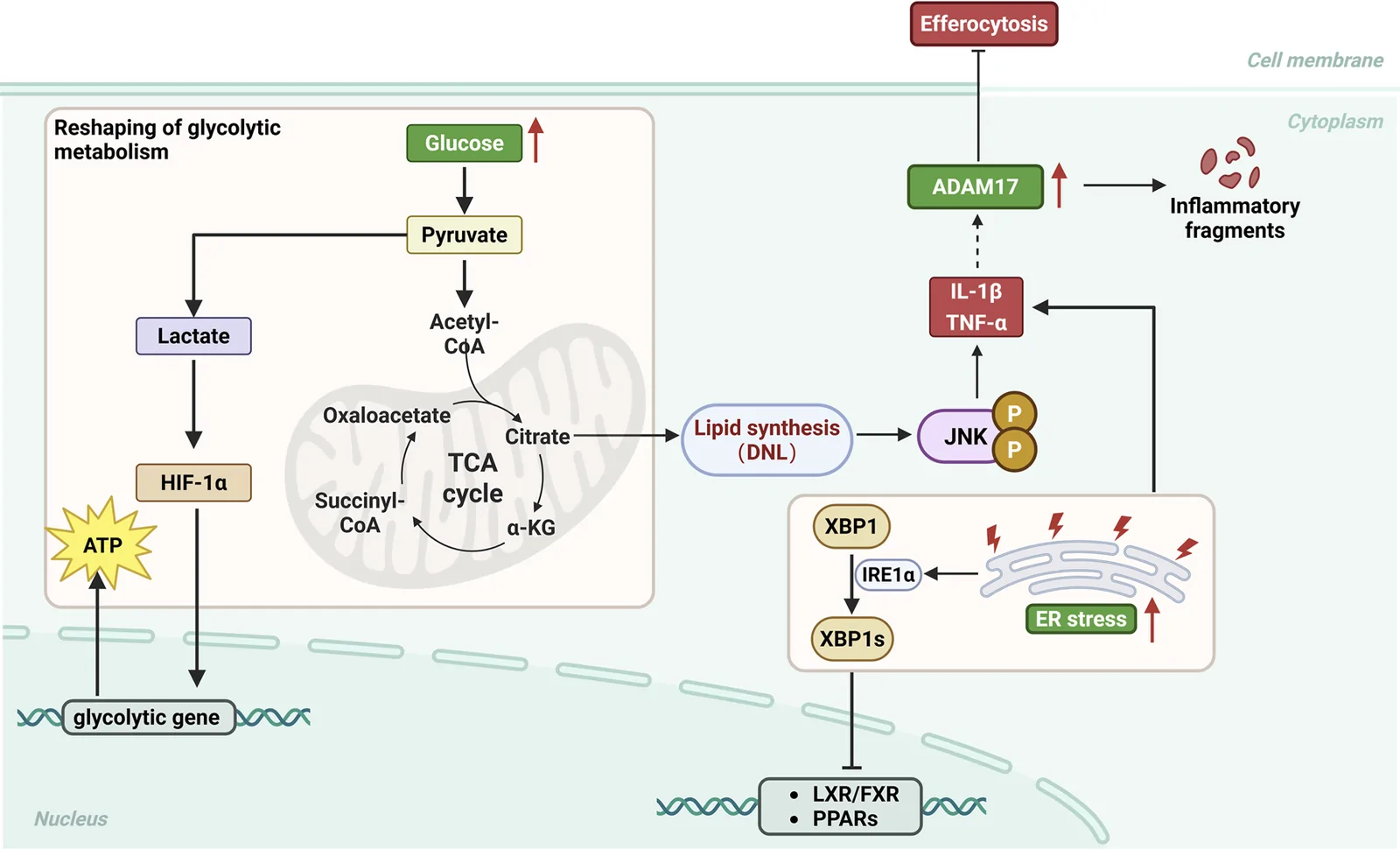
Metabolic and functional regulatory mechanisms of HSCs in the process of liver fibrosis. In the liver fibrosis microenvironment, glycol metabolism in macrophages is remodelled, glycolysis is increased, glucose is converted to pyruvate through glycolysis, and some of it is transformed into lactate, which activates the hypoxia-inducible factor HIF-1α, driving the expression of glycolytic genes and allowing macrophages to rely on glycolysis for energy to adapt to the inflammatory environment. Moreover, pyruvate enters the tricarboxylic acid (TCA) cycle and *de novo* lipogenesis (DNL), providing lipids for lipid accumulation in macrophages, activating the p-JNK pathway, and inducing the release of the proinflammatory factors IL-1β and TNF-α, which directly drive inflammation. Metabolic abnormalities also trigger endoplasmic reticulum stress (ER stress), which activates the IRE1α enzyme, which splices XBP1 to generate the active form of XBP1s, further amplifying the inflammatory signal and inhibiting the normal function of the nuclear receptors LXR/FXR and PPARs, weakening anti-inflammatory regulation. In addition, inflammation induces an increase in ADAM17 enzyme expression, leading to a decrease in efferocytosis (the ability to clear apoptotic cells) and the accumulation of inflammatory debris, resulting in a vicious proinflammatory cycle. In summary, metabolic abnormalities lead to the activation of inflammatory pathways, weakening of anti-inflammatory regulation, and defects in efferocytosis, causing macrophages to sustain proinflammatory activation and promote the progression of liver fibrosis.

Activated HSCs (MFs) are the core effector cells of BA-associated liver fibrosis, promoting fibrosis progression through four interrelated mechanisms. First, they synthesize and secrete large amounts of ECM components-including collagen type I/III, fibronectin, and laminin-disrupting the balance between ECM synthesis and degradation, leading to excessive ECM deposition, fibrous septa formation, and disruption of normal liver architecture (Ramm et al., 1998; Kitto and Henderson, 2021). Second, they possess strong contractile capacity, constricting hepatic sinusoids and peribiliary tissues, aggravating intrahepatic circulation disorders and bile stasis, and further amplifying liver injury (Kitto and Henderson, 2021). Third, through autocrine/paracrine effects, they secrete pro-fibrotic factors such as TGF-β and PDGF, as well as pro-inflammatory factors such as IL-6, which maintain their own continuous activation while regulating the functions of BECs and immune cells, participating in a vicious cycle of “injury-inflammation-fibrosis” (Fabris et al., 2017; Udomsinprasert et al., 2022). Fourth, activated HSCs undergo significant metabolic reprogramming that supports their profibrotic phenotype (Horn and Tacke, 2024). In BA-relevant models, activated HSCs shift toward aerobic glycolysis (Warburg effect), relying on lactate production as a key energy source (Gajendiran et al., 2018; Chen et al., 2012) (Figure 3). Research by Chen et al. (2012) demonstrated that activated HSCs accumulate large amounts of intracellular lactate even with high expression of monocarboxylate transporter 4 (MCT4), and inhibiting lactate accumulation can promote the reversion of MFs to quiescent HSCs, indicating lactate’s critical role in HSC activation (Chen et al., 2012). While aerobic glycolysis dominates, oxidative phosphorylation also provides energy support, with increased mitochondrial number and activity in activated HSCs promoting reactive oxygen species (ROS) production (Hellerbrand et al., 1999; Liu and Desai, 2015). ROS form a positive feedback loop by activating TGF-β signaling and inducing hepatocyte damage, indirectly driving fibrosis progression (Jain et al., 2013). In terms of glucose metabolism, activated HSCs highly express glucose transporters (GLUT1, GLUT2) and key glycolytic enzymes (HK2, PFKFB3, PK) while downregulating gluconeogenesis-related proteins (PCK1, FBP1) (Lin and Chen, 2011; Chandrashekaran et al., 2016). This metabolic shift is regulated by the Hedgehog signaling pathway, which promotes glycolysis by inducing hypoxia-inducible factor 1α (HIF1α) expression (Mejias et al., 2020; Zheng et al., 2020). Protein metabolism also undergoes significant changes, with approximately 38% of differentially expressed genes in activated HSCs related to protein metabolism-particularly the glutamine catabolic pathway, which is significantly activated (Du et al., 2018). Glutaminase-1 (GLS-1), the rate-limiting enzyme of this pathway, is upregulated in activated HSCs and colocalizes with MFB markers (Du et al., 2020). Inhibiting glutamine metabolism blocks HSC activation, whereas glucose deprivation does not, indicating that glutamine is a key energy source for maintaining the MFB phenotype (Trivedi et al., 2021), a process regulated by YAP/TAZ downstream effectors of the Hedgehog pathway. Abnormal lipid metabolism is also an important feature, with acetyl-CoA carboxylase (ACC) inhibition reducing HSC activation markers and improving liver fibrosis (Lally et al., 2019; Bates et al., 2020).

From a clinical perspective, the degree of HSC activation is closely related to the grade of BA-associated liver fibrosis and patient prognosis (Ramm et al., 1998). The number of activated HSCs and the expression levels of fibrosis markers (such as α-SMA and collagen type I) serve as important indicators for evaluating the severity and progression of BA-associated liver fibrosis (Kitto and Henderson, 2021). Targeting HSC activation has become a core strategy for anti-fibrotic treatment of BA. Inhibiting HSC-to-MF transformation by blocking key signaling pathways such as TGF-β and PDGF, or promoting apoptosis of activated HSCs and reversing their activated state, can effectively reduce ECM deposition and delay the progression of BA-associated liver fibrosis (Kitto and Henderson, 2021). Even after KPE, targeted inhibition of HSC activation can effectively delay postoperative fibrosis progression, providing a new intervention direction for extending native liver survival and reducing the need for liver transplantation.

#### 2.2.3 Immune cells

BA-associated liver fibrosis is a typical inflammation-driven fibrosis, and its pathological process is closely related to the abnormal activation, infiltration, and dysfunction of intrahepatic immune cells (Bessho and Bezerra, 2011). As the core regulators of the inflammatory microenvironment, immune cells are not only direct participants in bile duct injury but also promote the occurrence and progression of BA-associated liver fibrosis by releasing inflammatory factors, regulating the activation of HSCs, and participating in intercellular interactions to construct a pro-fibrotic microenvironment (Li et al., 2026). The types of abnormally activated immune cells in BA liver tissues are complex, mainly including macrophages, T lymphocytes, natural killer (NK) cells, and neutrophils. Each type of cell synergistically participates in fibrosis regulation through unique activation phenotypes and mechanisms, forming a complex immune regulatory network (Li et al., 2026; Bessho and Bezerra, 2011; Miller et al., 2024).

Macrophages are the most abundant infiltrating immune cells in BA liver tissues and also the key bridge connecting inflammation and fibrosis, mainly divided into liver-resident Kupffer cells (KCs) and peripheral monocyte-derived macrophages83. Under physiological conditions, KCs are in a quiescent state, mainly functioning to clear foreign bodies and maintain liver immune homeostasis. However, in the pathological microenvironment of BA, KCs are abnormally activated, and a large number of peripheral monocytes are recruited to the liver tissue and differentiate into macrophages (Miller et al., 2024). Both of them polarize into pro-inflammatory M1 type and pro-fibrotic M2 type (Ceci et al., 2024). Among them, scar-associated macrophages (SAMs) are the core subpopulation in the fibrotic microenvironment and play an important regulatory role in fibrosis progression (Li et al., 2026). M1-type macrophages mainly exert damaging effects by releasing pro-inflammatory factors. The TNF-α and IL-1β secreted by them can directly aggravate BECs injury, amplify intrahepatic inflammatory responses, and indirectly promote HSCs activation by activating the NF-κB and MAPK signaling pathways (Miller et al., 2024). M2-type macrophages and SAMs mainly have pro-fibrotic functions. They directly activate the transformation of HSCs into MFs and enhance the ECM synthesis capacity by secreting cytokines such as TGF-β, PDGF, and CCL2 (Miller et al., 2024). Meanwhile, these macrophages can maintain the stability of the fibrotic microenvironment by clearing apoptotic cells in the liver, avoid excessive uncontrolled inflammatory responses, and indirectly promote the continuous progression of fibrosis (Li et al., 2026). Single-cell sequencing studies have confirmed that CD14^+^, CD16^+^ intermediate monocytes and FCN3+ neutrophils are significantly enriched in BA fibrotic liver, which can further regulate myofibroblast proliferation and fibrosis progression through signaling axes such as FGF-FGFR and TNFRSF1A/B-GRN (Li et al., 2026).

Abnormal activation of T lymphocytes and immune imbalance are important regulatory factors in BA-associated liver fibrosis, among which the functional abnormalities of CD8^+^ T cells and regulatory T cells (Tregs) are the most critical83. CD8^+^ T cells are extensively infiltrated in BA liver tissues. As the main cytotoxic T cells, they can directly attack injured BECs and hepatocytes by releasing cytotoxic substances such as perforin and granzyme, thereby aggravating liver tissue damage (Miller et al., 2024). At the same time, the cytokines such as interferon-γ (IFN-γ) and TNF-α secreted by them can amplify inflammatory responses and promote HSCs activation and ECM deposition (Bessho and Bezerra, 2011). It is worth noting that even if bile drainage is successfully restored through KPE, the abundance of CD8^+^ T cells in liver remains persistently elevated, which is closely related to the progression of postoperative fibrosis and is an important factor affecting the prognosis of children (Lui, 2025). As a T cell subpopulation with immunosuppressive functions, Tregs can inhibit excessive inflammatory responses and maintain liver immune tolerance by secreting inhibitory cytokines such as IL-10 and TGF-β1 under physiological conditions (Miller et al., 2024). However, in BA, Treg function is significantly abnormal, and its immunosuppressive capacity is reduced, which cannot effectively inhibit the activation of pro-inflammatory cells such as CD8^+^ T cells and macrophages, leading to intrahepatic immune imbalance, continuous amplification of inflammatory responses, and further aggravation of bile duct injury and fibrosis. In addition, the imbalance of helper T cell (Th) subsets also participates in the regulation of BA fibrosis. The pro-inflammatory factors secreted by Th1 cells and the pro-fibrotic factors secreted by Th2 cells act synergistically to promote the progression of inflammation and fibrosis (Bessho and Bezerra, 2011).

NK cells exhibit a dual role in BA-associated liver fibrosis, but their overall effect is mainly pro-fibrotic. Under physiological conditions, NK cells can induce apoptosis of activated HSCs by releasing perforin and granzyme, exerting an anti-fibrotic effect. However, in the pathological microenvironment of BA, NK cell function is significantly inhibited, its anti-fibrotic effect is weakened, and it can secrete pro-inflammatory factors such as IFN-γ and TNF-α to amplify inflammatory responses, indirectly promoting HSCs activation and fibrosis progression (Miller et al., 2024). Studies have shown that the number of infiltrated NK cells in BA liver is reduced and their activity is decreased, and the degree of functional inhibition is positively correlated with fibrosis grade, suggesting that abnormal NK cell function may be an important driving factor for the progression of BA fibrosis (Miller et al., 2024).

Neutrophils are the main immune cells infiltrated in the early stage of BA-associated liver fibrosis, and they can accumulate in large numbers around the portal area and bile ducts of liver in the early stage of the disease (Miller et al., 2024). Activated neutrophils can directly damage BECs and hepatocytes and destroy the integrity of bile duct structure by releasing substances such as ROS and matrix metalloproteinases (MMPs) (Miller et al., 2024). At the same time, the chemokines such as CXCL8 and CXCL1 secreted by them can further recruit immune cells such as macrophages and T lymphocytes to infiltrate liver tissues, initiate inflammatory cascade reactions, and lay the foundation for the occurrence of fibrosis (Miller et al., 2024). In addition, the pro-inflammatory factors released by neutrophils can also directly activate HSCs and accelerate the progression of fibrosis. The degree of their infiltration is closely related to early liver injury and the progression rate of fibrosis in BA (Li et al., 2026).

In summary, immune cells play a core regulatory role in BA-associated liver fibrosis through abnormal activation, phenotypic polarization, and intercellular interactions: macrophages connect inflammation and fibrosis, T lymphocytes mediate immune imbalance and tissue damage, NK cell dysfunction weakens the anti-fibrotic effect, and neutrophils initiate early inflammatory responses. All types of cells act synergistically to form a complex regulatory network with BECs and HSCs, jointly promoting the vicious cycle of inflammation-fibrosis.

#### 2.2.4 Portal fibroblasts (PFs)

PFs are resident mesenchymal cells located in the portal region of the liver, mainly distributed around the bile ducts and the walls of portal blood vessels in the portal area (Zagory et al., 2015). They are indispensable key participating cells in the progression of BA-associated liver fibrosis, and play a unique and important role especially in the occurrence and development of biliary-derived fibrosis (Zagory et al., 2015). Compared with HSCs, PFs are more sensitive to cholestatic injury and are the main cellular source of periportal fibrosis (Dranoff and Wells, 2010). After abnormal activation, PFs can interact synergistically with BECs, HSCs, and immune cells to jointly promote the progression of BA-associated liver fibrosis, forming an important component of the “injury-inflammation-fibrosis” regulatory network.

Under physiological conditions, PFs are in a quiescent state with a spindle-shaped morphology. Their main functions are to maintain the structural integrity of the portal region and synthesize a small amount of ECM to maintain the normal homeostasis of the liver portal area (Zagory et al., 2015). At this stage, PFs do not express or weakly express fibrosis-related markers and play an auxiliary role in maintaining the normal physiological functions of the liver (Koyama et al., 2017). However, in the pathological microenvironment of BA, quiescent PFs are abnormally activated and gradually transform into MFs under the influence of various factors such as cholestasis, inflammatory stimulation, and intercellular signal regulation. Their phenotypes and functions undergo significant changes, making them the core effector cells driving periportal fibrosis.

The abnormal activation of PFs in BA is regulated by multiple signals, among which the main inducing factors are pro-fibrotic factors secreted by activated BECs and immune cells, as well as the toxic effect of accumulated bile acids (Fabris et al., 2017). The TGF-β and PDGF secreted by activated BECs are key factors for activating PFs. TGF-β can directly upregulate the expression of fibrosis-related genes such as collagen type I/III, fibronectin, and CTGF in PFs by activating the Smad2/3 signaling pathway, thereby promoting the transformation of PFs into MFs (Fuji et al., 2021). PDGF mainly enhances the proliferation and migration abilities of PFs by activating the PI3K/Akt signaling pathway, promoting their aggregation to bile duct injury sites and amplifying the fibrotic effect (Fuji et al., 2021). Pro-inflammatory and pro-fibrotic factors secreted by immune cells also participate in the regulation of PF activation. The TNF-α, IL-6, IL-1β, etc., secreted by a large number of infiltrating macrophages and T lymphocytes in BA liver tissues, can activate the NF-κB and MAPK signaling pathways, and act synergistically with TGF-β to accelerate PFs activation and phenotypic transformation (Li X. et al., 2025). In addition, bile acid accumulation caused by impaired bile excretion in BA can induce abnormal activation of PFs either by directly stimulating bile acid receptors (such as TGR5) on the surface of PFs or indirectly by activating BECs and macrophages to release pro-fibrotic factors, forming a positive cycle of cholestasis-PF activation-periportal fibrosis (Nyhol et al., 2024).

Activated PFs (i.e., MFs) participate in the progression of BA-associated liver fibrosis mainly through three aspects: First, they synthesize and secrete a large amount of ECM. The ECM components such as collagen type I, collagen type III, and laminin secreted by them are mainly deposited in the periportal and fibrous septal regions, leading to aggravated periportal fibrosis, further formation of fibrous cords, damage to the structure of the liver portal area, and obstruction of bile excretion and intrahepatic blood circulation (Koyama et al., 2017); Second, through autocrine/paracrine effects, they secrete pro-fibrotic factors such as TGF-β and PDGF, which not only maintain their own continuous activation but also regulate the functions of HSCs and BECs, promoting HSCs activation and BECs EMT, and further amplifying the fibrotic effect (Karin et al., 2016); Third, they participate in the construction of a pro-fibrotic microenvironment, and transmit injury and fibrosis signals through direct interactions with immune cells, HSCs, and BECs, synergistically promoting the vicious cycle of inflammation-fibrosis (Li X. et al., 2025).

Compared with HSCs, PFs have unique advantages and functional characteristics in BA fibrosis: PFs are mainly distributed in the portal area, while the core of BA injury is portal area bile duct inflammation and obstruction. Therefore, PFs can respond more directly to bile duct injury signals, activate rapidly, and participate in periportal fibrosis (Zagory et al., 2015). At the same time, PFs are significantly more sensitive to bile acids than HSCs and are activated earlier in cholestatic injury, making them one of the important initiators of early BA fibrosis (Koyama et al., 2017). Studies have confirmed that the degree of PF activation in BA liver tissues is closely related to the grade of periportal fibrosis, and the number of PFs and the expression levels of fibrosis markers (such as α-SMA and collagen type I) can be used as important indicators to evaluate the severity of BA-associated liver fibrosis (Zagory et al., 2015).

### 2.3 BA fibrosis-related signalling pathways

**FIGURE 4:**
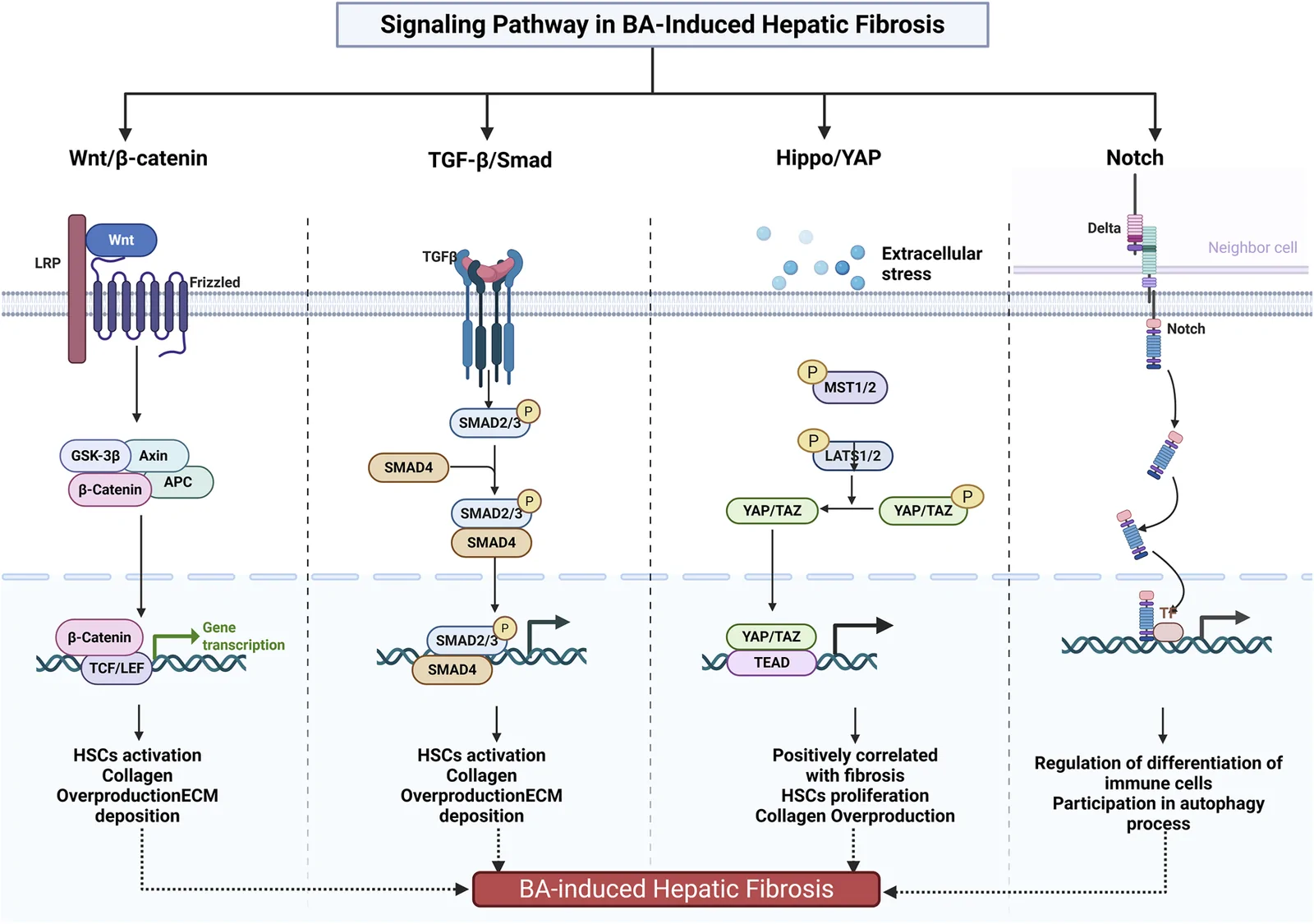
The relevant signalling pathways of BA-related liver fibrosis. We present two aspects: core signalling pathway analysis and potential therapeutic strategies. In the core signalling pathway analysis, four pathways were identified: the TGF-β/Smad, Hippo/YAP, Wnt, and Notch pathways. In the TGF-β/Smad pathway, the expression of TGF-β increases, activating Smad signalling and promoting the activation of hepatic stellate cells and collagen deposition, ultimately leading to excessive accumulation of ECM. In the Hippo/YAP pathway, YAP expression is upregulated and positively correlated with fibrosis, which can promote the proliferation of hepatic stellate cells and collagen synthesis. The Wnt pathway affects ECM deposition by regulating the activation of hepatic stellate cells. Activation of the Notch pathway signals promotes fibrosis and involves the regulation of immune cell differentiation and participation in the autophagy process.

BA-associated liver fibrosis is driven by a complex signaling network that coordinates hepatic stellate cells, biliary epithelial cells, and immune cells, leading to excessive extracellular matrix deposition and progressive liver damage. Rather than operating in isolation, these pathways engage in extensive crosstalk and feedback regulation, forming an integrated pro-fibrotic network. Based on robust evidence, these pathways are central drivers: Wnt/β-catenin, TGF-β/Smad, Hippo/YAP, and Notch (Figure 4). These pathways share downstream effectors and mutually reinforce each other, creating a self-perpetuating fibrotic milieu refractory to single-agent therapies.

#### 2.3.1 Wnt/β-catenin

The canonical β-catenin-dependent Wnt pathway is the important subtype driving BA-associated fibrosis (Wang et al., 2024; Tian et al., 2022). Under physiological conditions, the pathway remains quiescent in healthy liver: cytoplasmic β-catenin is incorporated into a degradation complex (APC, AXIN, GSK3, CK1), followed by phosphorylation and ubiquitin-proteasomal degradation to maintain low intracellular levels. Upon activation, Wnt ligands bind to membrane Frizzled (FZD) and LRP5/6 receptors, recruit Dishevelled (Dvl) to disrupt the degradation complex, block β-catenin breakdown, and trigger its cytoplasmic accumulation and nuclear translocation. Nuclear β-catenin binds to the TCF/LEF1 transcription complex to initiate transcription of downstream targets related to cell proliferation, EMT, and ECM deposition (Tian et al., 2022). LGR5 binding to R-Spondin further amplifies pathway activation by enhancing cellular sensitivity to Wnt ligands (Nusse and Clevers, 2017; de Lau et al., 2014).

Physiologically, this pathway regulates biliary development and damage repair by promoting BECs proliferation. In BA, its persistent excessive activation drives abnormal BECs proliferation, DR, EMT, and fibrosis-related gene expression, shifting repair toward fibrogenesis and aggravating bile duct obstruction and liver fibrosis (Tian et al., 2022). Mechanistically, TGF-β1-induced SULT2B1 overexpression activates the pathway and upregulates its downstream target MMP7 (mainly derived from BECs), which mediates BECs EMT and fibrosis progression; silencing SULT2B1 blocks this profibrotic cascade (Yang et al., 2025). Serum MMP7 also serves as a potential biomarker for BA diagnosis, monitoring, and prognosis, as its levels are significantly elevated in BA patients and correlate with liver stiffness (Yang et al., 2025).

In BA liver tissues, activated Wnt/β-catenin signaling drives HSCs activation via dual mechanisms: it directly initiates transcription of HSCs activation markers (α-SMA, collagen I) via nuclear β-catenin/TCF/LEF1 binding to promote HSCs proliferation, activation, and ECM synthesis (Schuppan, 2015); it also indirectly enhances HSCs activation by upregulating profibrotic cytokines (TGF-β1, PDGF), forming a profibrotic positive feedback loop. Clinical studies confirm that hepatic β-catenin expression in BA children positively correlates with α-SMA levels and liver fibrosis severity (Schuppan, 2015).

#### 2.3.2 TGF-β/Smad

The TGF-β/Smad pathway is the central profibrotic cascade in BA. Germline variations of its related genes are linked to biliary developmental abnormalities, ductal plate malformations, and BA pathogenesis (Ayabe et al., 2025). Studies using BA patient-derived multi-compartment biliary organoids (MBOs) reveal that epithelial layer defects and EMT in diseased organoids are tightly correlated with TGF-β/Smad2/3 pathway activation; inhibiting TGF-β signaling effectively suppresses EMT, promotes biliary epithelial development, and alleviates experimental BA phenotypes in neonatal mice, confirming the critical role of TGF-β-dependent EMT in BA biliary damage and fibrosis.

Clinically, TGF-β1 is significantly upregulated in BA children’s liver tissues, mainly concentrated in DR areas and surrounding inflammatory cells (Iordanskaia et al., 2013). It directly induces BECs EMT via the Smad2/3 pathway to accelerate ECM deposition and liver fibrosis48. For HSCs, activated TGF-β binds to HSCs surface receptors, triggers Smad2/3 signaling, and initiates transcription of activation-related targets (α-SMA, collagen I) to promote HSCs proliferation, activation, and ECM synthesis (Ceci et al., 2024; Schuppan, 2015). It also indirectly enhances HSCs activation via upregulating other profibrotic cytokines (e.g., PDGF), forming a profibrotic positive feedback loop. Clinical data confirm that hepatic TGF-β1 expression in BA children positively correlates with α-SMA levels and liver fibrosis severity (Ceci et al., 2024; Schuppan, 2015).

#### 2.3.3 Hippo/YAP

The Hippo/YAP pathway is a key regulator of DR, with its aberrant inactivation (leading to Yes-associated protein, YAP hyperactivation) driving excessive DR and BA-associated fibrosis (Molina et al., 2022). Clinically, YAP is significantly upregulated in BA children’s liver tissues, mainly localized to BECs and DR areas, with its expression positively correlated with DR severity and liver fibrosis grade. In the biliatresone-induced BA mouse model, Hippo pathway inhibition causes abnormal YAP accumulation and activation in BECs, driving excessive BECs proliferation and DR; conversely, Hippo activation and YAP inhibition significantly reduce BECs proliferation, alleviate DR, and attenuate liver fibrosis (Fried et al., 2025). YAP promotes BECs proliferation via upregulating cell cycle regulators (Cyclin D1, CDK4) and inhibits cholangiocyte apoptosis, aggravating biliary structural damage (Fabris et al., 2017; Xie et al., 2025). YAP also directly initiates transcription of EMT transcription factors (Twist1, Snail) via binding to TEA domain transcription factors (TEADs), downregulating E-cadherin and upregulating N-cadherin/vimentin to induce BECs EMT (Xie et al., 2025). It synergizes with the TGF-β/Smad pathway to amplify TGF-β-induced EMT, and YAP inhibition in BA patient-derived biliary organoids significantly suppresses EMT, restores biliary epithelial phenotype, and reduces fibrotic phenotypes.

In BA, hyperactivated YAP drives HSCs activation via dual mechanisms: it directly binds to TEADs in HSCs to initiate transcription of activation-related targets (α-SMA, collagen I, CTGF), promoting HSCs proliferation, activation, and ECM synthesis (Jiang et al., 2025); it also indirectly enhances HSCs activation via upregulating profibrotic cytokines (TGF-β1, PDGF), forming a profibrotic positive feedback loop. *In vitro* studies confirm that YAP silencing inhibits HSCs activation and ECM secretion, while YAP overexpression enhances HSCs profibrotic activity. Clinical data show that hepatic YAP expression in BA children positively correlates with α-SMA levels and liver fibrosis severity (Zhubanchaliyev et al., 2016).

#### 2.3.4 Notch

The Notch pathway is another key regulator of DR, with its aberrant activation driving excessive DR, biliary injury, and fibrosis in BA (Le et al., 2015). Clinically, Notch pathway-related molecules (Jag1, Notch2, Notch intracellular domain [NICD], downstream target Hes1) are significantly upregulated in BA children’s liver tissues, mainly localized to DR areas, with expression levels positively correlated with DR severity and liver fibrosis grade (Le et al., 2015). Mechanistically, activated Notch signaling promotes abnormal BECs proliferation via upregulating cell cycle regulators (Cyclin D1, CDK2), inhibits BECs apoptosis, and drives BECs secretion of profibrotic cytokines to recruit inflammatory cells and HSCs, aggravating biliary damage and fibrosis (Geisler and Strazzabosco, 2015).

NICD released upon pathway activation directly initiates transcription of EMT transcription factors (Snail1, Twist) in the nucleus, downregulating E-cadherin and upregulating N-cadherin/vimentin to induce cholangiocyte EMT (Jin et al., 2020). It synergizes with the TGF-β/Smad pathway to amplify TGF-β-induced EMT, and Notch inhibition in BA patient-derived biliary organoids significantly suppresses EMT, restores biliary epithelial phenotype, and reduces fibrotic phenotypes (Bakalenko et al., 2024).

In BA, activated Notch signaling drives HSCs activation via dual mechanisms: Notch ligands (e.g., Jag1) bind to HSCs surface Notch receptors, trigger NICD release and nuclear translocation, where NICD binds to RBP-Jκ to initiate transcription of activation-related targets (α-SMA, collagen I, CTGF), promoting HSCs proliferation, activation, and ECM synthesis; it also indirectly enhances HSCs activation via upregulating profibrotic cytokines (TGF-β1, IL-13), forming a profibrotic positive feedback loop (Le et al., 2015). *In vitro* studies confirm that silencing Notch1/Jag1 inhibits HSCs activation, while Notch1 overexpression enhances HSCs profibrotic activity. Clinical data show that hepatic Notch pathway molecule expression in BA children positively correlates with α-SMA levels and liver fibrosis severity.

Additionally, the Notch pathway shapes the BA inflammatory microenvironment to indirectly promote fibrosis: its activation positively correlates with inflammatory cell (macrophage, lymphocyte) infiltration in BA liver tissues (Bansal et al., 2015); it drives M1 macrophage polarization and pro-inflammatory cytokine (TNF-α, IL-6, IL-1β) secretion, which further activates Notch and other profibrotic pathways (e.g., TGF-β/Smad), forming an inflammation-fibrosis positive feedback loop (Yang et al., 2023). It also regulates lymphocyte proliferation/differentiation to sustain inflammatory responses, and mediates fibrosis via the IL-13/TGF-β1 axis under *Klebsiella pneumoniae* infection (Meng et al., 2021).

#### 2.3.5 Other important signaling pathways

The Hedgehog (Hh) pathway is a key profibrotic cascade in BA, mainly driving fibrosis via inducing BECs EMT and immature BECs proliferation (Kumar et al., 2021; Omenetti et al., 2011b). Its core components include ligands (e.g., Sonic Hedgehog, SHH), receptors, and downstream transcription factors (GLI1, GLI2). Clinically, the Hh pathway is significantly activated in BA children’s liver tissues: SHH is mainly expressed in BECs cytoplasm, while GLI1/GLI2 are highly expressed in BECs nuclei, with SHH and GLI2 mRNA/protein levels significantly higher in BA patients than in those with congenital biliary dilatation (CBD). *In vitro*, recombinant SHH protein activates the Hh pathway to downregulate epithelial marker E-cadherin and upregulate mesenchymal marker N-cadherin in BECs, inducing EMT; conversely, Hh inhibitor Cyclopamine significantly suppresses this EMT process (Omenetti et al., 2011b).

The PI3K/Akt pathway mainly regulates BA liver fibrosis via promoting HSCs activation and ECM deposition, with its activation closely linked to aberrant fibronectin 1 (FN1) expression (Liu et al., 2013). FN1, a core profibrotic gene, is highly expressed in BA liver tissues, with its levels positively correlated with liver fibrosis severity. Mechanistically, FN1 acts as an upstream regulator to activate PI3K, which catalyzes PIP3 generation to recruit and phosphorylate/activate Akt (Liu et al., 2013). Activated Akt promotes HSCs activation, proliferation, and anti-apoptosis via phosphorylating downstream targets, and upregulates ECM-related gene expression (Sharip and Kunz, 2025). Clinical studies confirm that PI3K and p-Akt expression in BA liver tissues positively correlates with FN1 and α-SMA levels. Additionally, the PI3K/Akt pathway crosstalks with the TGF-β/Smad pathway to amplify TGF-β′s profibrotic effect (Liu et al., 2013).

#### 2.3.6 Signal pathway interactions in BA fibrosis

BA-associated fibrosis progression is not driven by isolated single pathways; instead, core profibrotic cascades form a tightly interconnected regulatory network via upstream-downstream regulation, cross-activation, and synergistic amplification (Zhan et al., 2024a). The TGF-β pathway serves as the central hub with extensive crosstalk with other cascades, while close regulatory interactions also exist between the Notch, Hh, Wnt, and Hippo pathways, collectively driving fibrogenesis.

TGF-β and Hh pathways: The two pathways exhibit synergistic activation to promote EMT and HSC activation. Hh effector GLI2 directly binds to the TGF-β1 promoter to enhance its transcription, while TGF-β1 upregulates Hh ligand SHH expression via Smad3 signaling, forming a profibrotic positive feedback loop. Clinical studies confirm that SHH, GLI2, and TGF-β1 are significantly elevated and positively correlated in BA liver tissues (Fabris et al., 2016).

TGF-β and Notch pathways: The two pathways exhibit bidirectional cross-amplification to regulate cholangiocyte phenotype and inflammatory responses. Excessive Notch activation indirectly activates the TGF-β pathway via upregulating inflammatory cytokines (e.g., IL-6), while activated TGF-β signaling upregulates Notch1 and Jagged1 expression via Smad signaling, further enhancing Notch pathway activation. Germline variations in both pathways also jointly participate in BA pathogenesis initiation (Le et al., 2015; Bansal et al., 2015; Fabris et al., 2016).

Wnt and Hippo pathways: The two pathways exhibit close cross-regulation in cholangiocyte injury and fibrotic repair. In BA cholangiocyte injury models, Hippo components Pard6g and Amotl1 act as upstream regulators of Wnt pathway activation, while activated Wnt signaling regulates Hippo downstream effectors via β-catenin to modulate cholangiocyte proliferation and apoptosis; imbalanced regulation between the two pathways aggravates cholangiocyte injury and fibrosis. The Wnt pathway also synergizes with the TGF-β pathway via upregulating TGF-β1 expression to amplify profibrotic effects (Li et al., 2014; Sha et al., 2024).

### 2.4 Epigenetic regulation

#### 2.4.1 DNA methylation

Abnormal DNA methylation is one of the most important epigenetic abnormalities in BA-associated fibrosis, which is mainly characterized by hypomethylation (abnormal activation) of pro-fibrotic genes and hypermethylation (abnormal silencing) of anti-fibrotic genes (Lyu S. Y. et al., 2023). It drives the progression of liver fibrosis by regulating the imbalance of gene expression (Aseem and Huebert, 2019). Clinical studies have shown that the expression levels of DNA methyltransferases (DNMT1, DNMT3A, DNMT3B) are significantly upregulated in the liver tissues of children with BA, and are positively correlated with the degree of liver fibrosis, suggesting that abnormal DNA methylation regulation is involved in the occurrence and development of BA-associated fibrosis (Yang et al., 2018). In terms of pro-fibrotic gene regulation, TGF-β1, as a core pro-fibrotic factor, shows hypomethylation at the CpG sites in its gene promoter region, leading to abnormal high expression of the TGF-β1 gene (Perakakis et al., 2020). This further activates the TGF-β/Smad signaling pathway, promoting HSCs activation, EMT, and ECM deposition (Lyu S. Y. et al., 2023). In addition, hypomethylation of the promoter region of the TIMP-1 gene can lead to its increased expression, which inhibits ECM degradation and further aggravates excessive ECM deposition (Barcena-Varela et al., 2019). In terms of anti-fibrotic gene regulation, peroxisome proliferator-activated receptor γ (PPARγ), a key anti-fibrotic gene, exhibits hypermethylation in its promoter region, resulting in silencing of the PPARγ gene (Aseem and Huebert, 2019). This prevents PPARγ from exerting its functions of inhibiting HSCs activation and reducing ECM synthesis, thereby accelerating the progression of liver fibrosis. Studies have confirmed that treatment of BA-related cell models with DNA methyltransferase inhibitors (such as 5-azacytidine) can significantly reverse the hypermethylation state of the PPARγ gene, restore its expression, and simultaneously inhibit the expression of pro-fibrotic genes such as TGF-β1 and TIMP-1 (Lyu et al., 2025). This treatment also reduces HSCs activation and the degree of fibrosis, suggesting that abnormal DNA methylation can serve as a potential therapeutic target for BA-associated fibrosis (Lyu S. Y. et al., 2023).

#### 2.4.2 Histone modification

Abnormal histone modification participates in the progression of BA-associated fibrosis mainly by altering chromatin status and regulating the transcriptional expression of pro-fibrotic related genes (Jang et al., 2025). Among them, the regulatory roles of abnormal histone acetylation and histone methylation are the most significant (Barcena-Varela et al., 2019). In terms of abnormal histone acetylation, the imbalance between histone deacetylases (HDACs) and histone acetyltransferases (HATs) is the core mechanism. In the liver tissues of children with BA, the expression levels of HDACs (mainly HDAC1, HDAC2, HDAC3) are significantly increased, while the expression levels of HATs are significantly decreased (Jang et al., 2025). This leads to a reduction in histone acetylation levels, resulting in a condensed chromatin state that inhibits the expression of anti-fibrotic genes and promotes the expression of pro-fibrotic genes. For example, HDACs can inhibit the transcription of anti-fibrotic genes and epithelial phenotype genes such as PPARγ and E-cadherin through deacetylation, while enhancing the transcription of pro-fibrotic genes such as TGF-β1 and α-SMA, thereby promoting HSCs activation, EMT, and the progression of liver fibrosis. *In vitro* experiments have confirmed that the use of HDAC inhibitors (such as trichostatin A and vorinostat) can significantly increase histone acetylation levels, restore the expression of anti-fibrotic genes, and inhibit the expression of pro-fibrotic genes (Theodoropoulou et al., 2024). This further inhibits HSCs activation, reduces ECM deposition, and alleviates BA-related fibrosis. In terms of abnormal histone methylation, it mainly involves abnormal modification sites such as histone H3K4me3 and H3K27me3 (Barcena-Varela et al., 2019). H3K4me3 is usually associated with gene activation, while H3K27me3 is usually associated with gene silencing (Jiang et al., 2021). Studies have found that in the liver tissues of children with BA, the level of H3K4me3 modification in the promoter regions of pro-fibrotic genes (such as TGF-β1 and α-SMA) is significantly increased, promoting their gene transcription (Barcena-Varela et al., 2019). In contrast, the level of H3K27me3 modification in the promoter region of anti-fibrotic genes (such as PPARγ) is significantly increased, inhibiting their gene transcription (Basta et al., 2023). This forms an imbalanced state where pro-fibrotic genes are activated and anti-fibrotic genes are silenced, accelerating the progression of liver fibrosis (Wilson et al., 2017). In addition, abnormal expression of histone methyltransferases and demethylases is also involved in this regulatory process, providing a new direction for the targeted treatment of BA-associated fibrosis (Jiang et al., 2021).

#### 2.4.3 Non-coding RNAs

As an important component of epigenetic regulation, non-coding RNAs (ncRNAs) are widely involved in various pathological links of BA-associated fibrosis through post-transcriptional regulation (Peng et al., 2025). Among them, the regulatory roles of microRNAs (miRNAs), long non-coding RNAs (lncRNAs), and circular RNAs (circRNAs) are the most clear, and the three interact with each other to form a complex regulatory network.

miRNAs are a class of small ncRNAs with a length of approximately 22 nucleotides. They regulate gene expression by binding to the 3′ untranslated region (3′UTR) of target mRNAs, inhibiting mRNA translation or promoting its degradation (Peng et al., 2025). Clinical studies have found that a variety of miRNAs are abnormally expressed in the liver tissues and serum of children with BA, among which miR-21, miR-199a-5p, and miR-146a are significantly highly expressed, while miR-122 and miR-378 are significantly lowly expressed (Yang et al., 2018). Highly expressed miR-21 can activate the PI3K/Akt and TGF-β/Smad signaling pathways by targeting and inhibiting the expression of anti-fibrotic molecules such as PTEN and Smad7, thereby promoting HSCs activation, EMT, and ECM deposition. miR-199a-5p can enhance HSCs activation and promote the progression of liver fibrosis by targeting and inhibiting the expression of PPARγ (Sha et al., 2024). Lowly expressed miR-122 can lead to abnormal high expression of its target genes (such as TGF-β1 and α-SMA), aggravating fibrosis (Peng et al., 2025). miR-378 exerts an anti-fibrotic effect by inhibiting the expression of pro-fibrotic genes, and its decreased expression weakens the anti-fibrotic effect (Wang et al., 2021).

lncRNAs are a class of ncRNAs with a length greater than 200 nucleotides. They are involved in gene expression regulation by adsorbing miRNAs, regulating histone modification, binding to DNA, and other methods (Peng et al., 2025). In the liver tissues of children with BA, lncRNA H19, lncRNA NEAT1, lncRNA MALAT1, etc., are significantly highly expressed, while lncRNA GAS5 is significantly lowly expressed (Zeybel et al., 2012). lncRNA H19 can promote the expression of pro-fibrotic genes by adsorbing miR-122 and relieving its inhibition on target genes, thereby accelerating HSCs activation and fibrosis progression (Donnelly et al., 2014). lncRNA NEAT1 can promote the transcription of pro-fibrotic genes by regulating the expression of HDACs and changing histone acetylation levels, thereby driving the progression of liver fibrosis (Chen, 2016). lncRNA GAS5 can inhibit the pro-fibrotic effect of miR-21 by adsorbing it, and its decreased expression leads to enhanced activity of miR-21 and aggravated fibrosis.

circRNAs are a class of circular ncRNAs with high stability and strong specificity, which mainly regulate gene expression by adsorbing miRNAs (as competing endogenous RNAs, ceRNAs) (Peng et al., 2025). Recent studies have found that circRNA 0001649, circRNA 42398, etc., are highly expressed in the liver tissues of children with BA, while circRNA 0005075 is lowly expressed (Lyu S. Y. et al., 2023). circRNA 0001649 can promote the expression of its target gene TGF-β1 by adsorbing miR-140-5p, activate the TGF-β/Smad signaling pathway, and drive HSCs activation and EMT (Omenetti et al., 2011b). circRNA 42398 can inhibit HSCs activation by regulating the TGF-β1/Smad signaling pathway, and its decreased expression weakens the anti-fibrotic effect, accelerating the progression of BA-associated fibrosis (Wang et al., 2017).

## 3 Advances in the imaging diagnosis of liver fibrosis

### 3.1 Ultrasound, contrast-enhanced ultrasound and ultrasound elastography technology

Conventional ultrasound plays an important role in the diagnosis of BA. Ultrasound examinations can quickly assess the structural condition of the bile ducts and the morphological characteristics of the liver, with common ultrasound diagnostic features including the triangular cord sign, absence of the gallbladder, and widening of the hepatic artery (Zhou and Zhou, 2021). However, ultrasound has certain limitations in assessing the degree of liver fibrosis, especially in cases of liver enlargement or complex liver lesions, where it may not provide sufficient information. Considering the limitations of conventional ultrasound, contrast-enhanced ultrasound (CEUS) has become an alternative method. Ultrasound imaging utilizes pulses of sound waves to generate visual representations of reflected signals from tissues. CEUS, also known as acoustic contrast, employs specialized contrast agents to enhance backscattered echoes in ultrasound imaging (Ying et al., 2025). In the liver, CEUS enables the detailed visualization of blood flow through the portal vein, hepatic artery, hepatic vein, and bile ducts, aiding in the diagnosis of liver parenchymal lesions (Beckmann and Simanowski, 2020). CEUS can be used to non-invasively evaluate the extent of liver fibrosis (Ferraioli et al., 2018). Compared to conventional methods, CEUS offers notable advantages, including high safety, convenience, accuracy, and cost-effectiveness. But, its application requires caution due to the high technical proficiency demanded of operators, time-sensitive imaging processes, and susceptibility to visual interference.

Ultrasound elastography technology is a noninvasive imaging technique based on conventional ultrasound that has made significant progress in the diagnosis of liver diseases in recent years. In particular, in the staging of liver fibrosis related to BA, 2D-SWE has shown high diagnostic value. Studies have indicated that 2D-SWE has good sensitivity and specificity, effectively distinguishing different degrees of liver fibrosis. The cut-off values to predict different degrees of liver fibrosis shown in Table 1. According to one study, the sensitivity of 2D-SWE reached 83%, with a specificity of 70%, indicating significant advantages in the early identification of liver fibrosis (Lyu H. et al., 2023). Preoperative and postoperative SWE values have also been fully validated for assessing the degree of liver fibrosis and predicting native liver outcomes (Yoon et al., 2023). Preoperative SWE can provide quantitative data on liver stiffness, which is crucial for formulating individualized surgical plans. Research shows that preoperative liver stiffness is significantly correlated with postoperative liver function recovery, with higher preoperative liver stiffness usually indicating poorer postoperative liver function outcomes. These findings suggest that SWE can serve as an important tool for assessing the liver condition of patients before they undergo Kasai surgery, helping doctors make more informed treatment decisions.

**TABLE 1:** The cut-off value of SWE and MRE in predicting liver fibrosis of BA.

Fibrosis stage	Cut-off value, kPa		
	S-SWE (Zhou et al., 2022)	T-SWE (Zhou et al., 2022)	MRE (Ozturk et al., 2022)
F0	<10.2	<8.7	<3.0
F1	10.2–13.0	8.7–8.9	3.0–3.5
F2	13.0–14.0	8.9–13.5	3.5–4.0
F3	14.0–27.3	13.5–14.7	4-5.0
F4	>27.3	>14.7	>5

S-SWE, supersonic shear wave elastography; T-SWE, toshiba shear wave elastography; MRE, magnetic resonance elastography.

Despite the advantages of SWE technology in the application of BA, its limitations cannot be ignored. First, the accuracy of SWE may be affected by factors such as the degree of patient obesity and the different locations of liver lesions. Additionally, SWE may not replace more direct assessment methods, such as liver biopsy, when evaluating the specific stage of liver fibrosis. Therefore, future research should focus on how to improve the accuracy and reliability of SWE, such as by combining it with other imaging examinations or biomarkers to increase its predictive ability (Russi et al., 2023). With the continuous development of technology, the application range of SWE may further expand, and the integration of artificial intelligence and machine learning algorithms may improve the precision of liver fibrosis assessments. Moreover, combining multimodal imaging techniques, such as ultrasound, computed tomography (CT), and magnetic resonance imaging (MRI), will help comprehensively assess liver health, providing more comprehensive and personalized treatment plans for patients with BA (Zeng X. et al., 2023).

### 3.2 MRI and other imaging techniques

MRI plays an important role in the diagnosis and assessment of liver fibrosis related to BA. MRI can not only provide structural information about the liver but also quantitatively assess liver cirrhosis. Studies have shown that combining MRI volumetric measurements (such as left liver lobe volume) with serological indicators (such as liver function indicators and tumor markers) can significantly increase the ability to predict liver cirrhosis. For example, a reduction in left liver lobe volume is closely related to the progression of liver fibrosis, providing important evidence for clinicians in assessing the severity of the disease. Additionally, magnetic resonance elastography (MRE), an emerging MRI technology, can noninvasively assess liver stiffness, providing new perspectives for the diagnosis of liver fibrosis (Gao et al., 2023).

### 3.3 Comparision between SWE, MRE, CEUS and ultrasound

In the previous section, we introduced the techniques for diagnosing liver fibrosis in BA, apart from biopsy. In this section, we will compare the advantages and disadvantages of these techniques. Biopsy can also effectively diagnose the damage to liver fibrosis. However, compared with biopsy, the other several techniques are all non-invasive and cause less harm to the patients. Therefore, we focused on comparing several non-invasive diagnostic techniques for liver fibrosis. For the average patients, these several techniques can provide relatively accurate diagnoses. However, for some special patients, such as those with enlarged livers, ultrasound can not provide sufficient information. Compared with MRE, ultrasound, including SWE and CEUS, has advantageous for pediatric patients, who are more susceptible to ionizing radiation and may require sedation or general anesthesia for MRI examinations (Squires et al., 2022). Another inherent advantages due to its portability, accessibility and cost-effective. In some diagnostic cases, such as focal liver lesion, CEUS is much more accurate than MRE (Ying et al., 2025). However, ultrasound, including SWE and CEUS, highly dependent on the operator’s proficiency and experience. In addition, CEUS is not accurate in diagnosing benign and malignant liver lesions compared with MRE.

## 4 Applications of serological and molecular biomarkers in liver fibrosis

### 4.1 Traditional serological indicators (APRI, FIB-4, etc.)

To monitor liver fibrosis caused by BAs, traditional serological indicators such as the aminotransferase (AST)/platelet ratio index (APRI) and FIB-4 score are widely used. These indicators, owing to their noninvasive and easily obtainable characteristics, allow clinicians to assess the liver health status of patients quickly in clinical practice. The advantage of the APRI lies in its ability to effectively reflect the degree of liver fibrosis, particularly in chronic liver disease patients, where it has been shown to have high accuracy. Multiple studies have indicated that the APRI is significantly correlated with the stage of liver fibrosis, especially in patients with chronic hepatitis B and nonalcoholic fatty liver disease (Åsberg et al., 2023). In BA, the optimal APRI cut-off value for diagnosing significant fibrosis (≥F2) ranges from 0.95 to 1.29, with sensitivity of 60.6%–88.6%, specificity of 76.0%–76.0%, and AUC of 0.65–0.75 (Nagi et al., 2023).

The FIB-4 index combines age, AST, aminotransferase (ALT), and platelet count, providing a more comprehensive assessment of liver fibrosis. Research has shown that the FIB-4 score also has different predictive abilities across different age groups. In patients without viral hepatitis infection, higher FIB-4 scores are positively correlated with the severity of liver fibrosis (Rigamonti et al., 2022; Wang C. X. et al., 2025). Specifically, in a study involving chronic liver disease patients, analysing changes in FIB-4 was able to better predict the risk of hepatocellular carcinoma. In BA, the optimal FIB-4 cut-off value for predicting severe fibrosis ranges from 9 (Bessho and Bezerra, 2011) (after multiplying by 100 to adjust for age in months) to 0.02 (unadjusted), with sensitivity of 82.6%–89.0%, specificity of 66.7%–70.0%, and AUC of 0.65–0.72146. These values are substantially different from the conventional cut-off of 1.3 used in adult chronic liver diseases, due to the unique age-related calculation and cholestatic pathophysiology in BA infants. Although the APRI and FIB-4 score demonstrate good sensitivity and specificity in assessing liver fibrosis, these indicators also have certain limitations. For example, the APRI may be influenced by fluctuations in platelet counts in some cases, affecting its accuracy. Additionally, the calculation of FIB-4 involves age factors, which may lead to different threshold settings for patients of different ages. These limitations suggest that caution should be exercised in interpreting these indicators in clinical applications (Sumida et al., 2021). In summary, the APRI and FIB-4, as traditional serological indicators, hold significant clinical value in monitoring liver fibrosis related to BA. They not only provide preliminary assessments of liver status but also assist clinicians in formulating corresponding treatment strategies. However, future research needs to further explore the performance of these indicators in patients with various liver diseases to improve their accuracy and reliability in assessing liver fibrosis. Additionally, combining results from other imaging and pathological examinations will help provide a more comprehensive diagnostic and treatment plan.

### 4.2 Emerging molecular biomarkers

**FIGURE 5:**
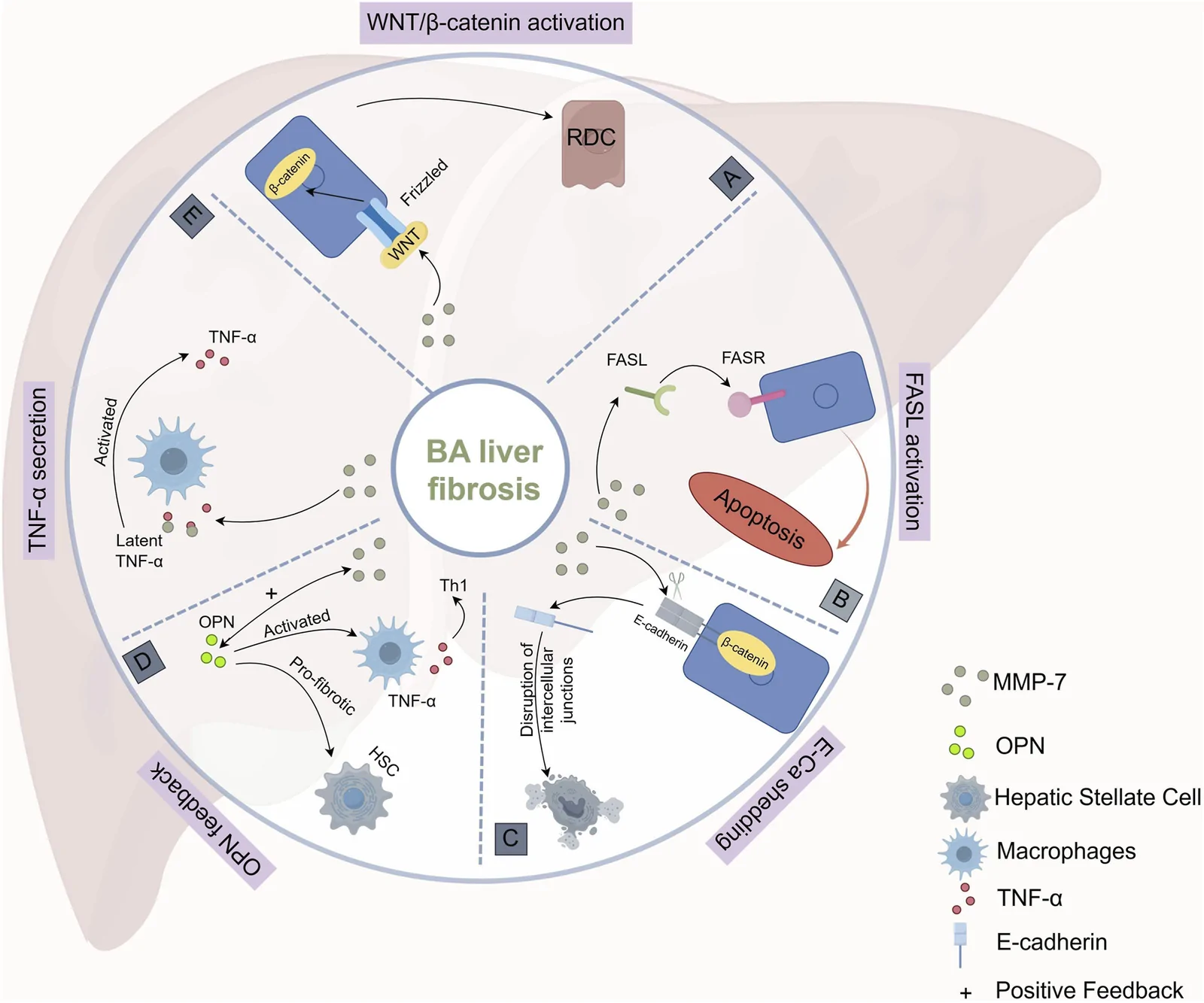
The mechanism by which MMP-7 promotes the progression of BA-related hepatic fibrosis. MMP-7 may promote the progression of BA-related hepatic fibrosis through the following mechanisms, either individually or synergistically. The main mechanisms include the following: **(A)** In terms of cell proliferation regulation, MMP-7 promotes the abnormal proliferation of BECs by activating the WNT/β-catenin signalling pathway, leading to the formation of reactive duct cells (RDCs), a process closely related to the repair and pathological changes in the biliary tract system. **(B)** In the apoptosis pathway, MMP-7 can activate Fas ligand (FASL), allowing it to specifically bind to the Fas receptor (FASR) on the cell surface, thereby initiating the apoptotic signalling cascade and ultimately leading to programmed cell death. **(C)** Regarding the impact on intercellular connections, MMP-7 can cause epithelial cadherin (E-cadherin) to be shed from the cell surface, an action that not only weakens the adhesive connections between cells but also further promotes the occurrence of apoptosis, forming a vicious cycle. **(D)** In the positive feedback regulatory mechanism, MMP-7 forms a mutually promoting regulatory loop with osteopontin (OPN). OPN, on the one hand, promotes the fibrotic process by activating hepatic stellate cells (HSCs), and on the other hand, it can stimulate macrophages to differentiate into the Th1 phenotype, thereby amplifying the immune response. **(E)** In the regulation of the inflammatory response, MMP-7 can activate macrophages, promoting their secretion of proinflammatory factors such as tumor necrosis factor α (TNF-α) and significantly increasing the intensity of local and systemic inflammatory responses.

In recent years, research on liver fibrosis related to BA has gradually revealed the potential diagnostic value of various emerging molecular biomarkers. Among them, serum matrix metalloproteinase-7 (MMP-7) has attracted widespread attention as an emerging liver fibrosis biomarker in BA patients (Pandurangi et al., 2024). Studies have shown that MMP-7 plays an important role in the pathogenesis of BA fibrosis, as its expression level is positively correlated with the degree of liver fibrosis (Jiang et al., 2024; Nomden et al., 2020) (Figure 5). A recent comprehensive meta-analysis of 17 studies (2,836 serum samples) demonstrated that serum MMP-7 has a pooled sensitivity of 93% (95% CI: 92%–94%), specificity of 85% (95% CI: 83%–87%), and AUC of 0.96 for diagnosing BA, though significant heterogeneity in cut-off values (0.49–67.4 ng/mL) was observed across studies (Wang H. et al., 2025). Age-stratified analysis suggested an optimal cut-off of 28.1 ng/mL for infants aged 0–30 days, with sensitivity of 86.4% and specificity of 95.5% (Wang H. et al., 2025). Therefore, MMP-7 may serve as a potential biomarker for assessing liver fibrosis in BA patients, helping clinicians make more accurate diagnostic and treatment decisions.

Additionally, amyloid precursor protein (APP) has recently been studied as an important biomarker for liver fibrosis. Research has shown that abnormal deposition of APP in the liver is closely related to the development of liver fibrosis, which primarily involves liver cell injury and inflammatory responses. High levels of APP can serve as diagnostic indicators for liver fibrosis, and they also show good prospects in predicting disease progression and treatment response (Cheng et al., 2023). Bile acids and their metabolites also play important roles in the process of liver fibrosis. Abnormal bile acid metabolism in BA patients may lead to liver cell injury and exacerbation of fibrosis. Studies have shown that the concentrations of bile acids and their metabolites are closely related to the severity of liver fibrosis (Karpen et al., 2020). These bile acid metabolites not only serve as biomarkers for liver fibrosis but also may provide new therapeutic targets for clinical practice. In summary, emerging molecular biomarkers such as MMP-7, APP, and bile acid metabolites hold significant clinical value in the diagnosis and treatment of liver fibrosis related to BA. In the future, further research on these biomarkers may provide more evidence for the early diagnosis and individualized treatment of BA patients, ultimately improving patient prognosis.

## 5 Applications of animal models and *in vitro* models in the study of the mechanisms of liver fibrosis

### 5.1 Toxin-induced models

In studying the pathogenesis of BA and its related liver fibrosis, toxin-induced models are particularly important. In recent studies, biliatresone has been used as the main toxin to induce BA in mouse models. Biliatresone is an effective chemical that disrupts the normal development and function of the bile ducts, leading to typical symptoms of BA and liver fibrosis in mice. In this model, changes in liver tissue are characterized by hepatocyte injury, bile duct fibrosis, and inflammatory responses in the liver, all of which are key pathological features of liver fibrosis related to BA (Zhu et al., 2023). In biliatresone-induced BA mouse models, researchers have shown that oxidative stress and depletion of glutathione play important roles in the development of liver fibrosis. Oxidative stress refers to elevated levels of ROS in the body, leading to cell damage and apoptosis. In this model, biliatresone-treated mice exhibit significant oxidative stress, accompanied by decreased activity of intracellular antioxidant enzymes (such as superoxide dismutase and glutathione peroxidase), resulting in hepatocyte injury and exacerbation of fibrosis (Gupta et al., 2024). Additionally, glutathione depletion is considered an important mechanism leading to liver fibrosis, with studies showing that reduced glutathione levels are directly related to oxidative stress levels in the liver, thereby affecting hepatocyte survival and function (Adeyemi et al., 2020). Furthermore, the establishment of the biliatresone model provides an experimental basis for exploring potential therapeutic strategies for BA (Schmidt et al., 2023). In this model, researchers have shown that intervention with antioxidants can effectively alleviate oxidative stress-induced damage to hepatocytes, thereby improving the survival rate and liver function of mice. These findings offer new ideas and directions for the development of clinical treatments for BA and liver fibrosis. Through further research on this model, scientists hope to reveal deeper mechanisms of liver fibrosis related to BA, providing new targets for clinical intervention.

### 5.2 Genetic gene knockout models

In studying the pathogenesis of BA and liver fibrosis, the PKD1L1 knockout mouse model serves as an important genetic knockout model, providing opportunities to explore ciliary function and pathological changes in the liver in depth. The PKD1L1 gene encodes a protein associated with ciliary growth and function, and its deletion is closely related to BA and subsequent liver fibrosis. PKD1L1 KO mice can use as the first genetically linked model of BA to study the pathophysiology of this serious and perplexing disorder, including the opportunity to identify rational therapeutic targets. Specifically, in the liver tissue of these mice, the expression of fibrosis-related genes (such as Tgfα and Pdgfc) is significantly elevated, whereas the expression of genes related to ciliary function (such as Gli1 and Gli2) is significantly reduced, suggesting that damage to ciliary function may play a central role in the pathogenesis of BA liver fibrosis. By studying this model, scientists can explore the role of cilia in liver development and function, as well as their potential impact on bile duct diseases. This model not only provides foundational data for studying the genetic mechanisms of BA but also lays the experimental groundwork for developing new therapeutic strategies. Further research may reveal how interventions targeting ciliary function can alleviate or reverse the progression of liver fibrosis, thus providing new treatment ideas for clinical practice. In summary, the PKD1L1 knockout mouse model is an important tool for studying BA and liver fibrosis, helping scientists better understand the mechanisms of fibrosis and providing new directions for future treatment strategies. Through in-depth research on this model, new biomarkers and therapeutic targets may be discovered, ultimately improving the prognosis and quality of life of patients with BA.

### 5.3 Rhesus rotavirus (RRV)-indeuced mouse model

RRV-induced BA mouse model is the most commonly used model for studying the pathogenesis of BA. This model encompasses most of the pathological features of BA, such as jaundice of the skin and mucosa, growth retardation and dark yellow urine. The histopathology shows severe liver inflammation and obstruction of the intrahepatic and extrahepatic bile ducts. Current research indicates that there is controversy regarding the reproducibility of this model for the pathological feature of liver fibrosis. Some study showed the histopathology shows obvious liver fibrosis in RRV-induced mouse model (Liu et al., 2024; Wang et al., 2020; Shen et al., 2014). In contrast, some study showed lack of liver fibrosis in this model (Wang X. et al., 2023; Luo et al., 2019). These studies indicate that liver fibrosis exhibits a reproducibility defect in this model, which also limits the research on liver fibrosis in BA using this model. In order to overcome this limitation, several studies have explored methods to establish a chronic fibrosis model base RRV-induced model. Wang X. et al. (2023) injected 5–10 µg of anti-Ly6G antibody four times with gaps of 2 days after each injection before RRV inoculation on the day of birth. They establish a chronic BA mouse model with typical liver fibrosis successfully. Mohanty et al. (2020) generated an RRV-TUCH rotavirus reassortant that when injected into newborn mice cause liver fibrosis with histopathological signs of inflammation/fibrosis and bile duct obstruction.

## 6 Treatment strategies for BA-associated liver fibrosis

### 6.1 Surgical intervention: KPE and its limitations

The KPE remains the first-line surgical intervention for BA, aimed at restoring bile flow by creating a Roux-en-Y anastomosis between the porta hepatis and the jejunum (Xing et al., 2024). Successful KPE-defined as achievement of jaundice clearance (total bilirubin <2 mg/dL)-can significantly delay the progression of liver fibrosis and improve native liver survival (Zhan et al., 2024b). However, the success of KPE is highly dependent on age at surgery, with outcomes significantly better when performed before 60–90 days of life (Tam et al., 2024). Even with successful KPE, liver fibrosis continues to progress in a substantial proportion of patients, and many eventually require liver transplantation (Sanada et al., 2022; de La Taille et al., 2021). For patients with end-stage liver fibrosis, liver transplantation remains the definitive treatment, with 5-year overall survival rates exceeding 80%–90% in contemporary series (Anouti et al., 2023; Shan et al., 2024). However, transplantation is limited by donor organ scarcity, the need for lifelong immunosuppression, and the morbidity associated with long-term immunosuppressive therapy (Shan et al., 2024).

### 6.2 Postoperative medical management and established adjunctive therapies

Following KPE, patients typically receive supportive medical therapy, including ursodeoxycholic acid (UDCA) (20 mg/kg/day) to enhance bile flow and antibiotic prophylaxis (e.g., trimethoprim-sulfamethoxazole) to prevent ascending cholangitis (Chen et al., 2025). However, the evidence for these interventions is mixed: a cohort study of 141 BA infants found that UDCA was not associated with improved outcomes and was associated with a range of hepatic and extrahepatic complications (Dong et al., 2022). The START trial, which evaluated high-dose corticosteroids post-KPE, failed to demonstrate a significant benefit in bile drainage at 6 months and was associated with earlier serious adverse events (Chen et al., 2025). These negative and mixed results underscore the urgent need for effective pharmacotherapies targeting the underlying fibrotic process.

### 6.3 Emerging pharmacological therapies

Currently, there are no specific drugs on the market for treating liver fibrosis of BA although there have been some attempts at research. For example, ligustrazine has received widespread attention as a potential antifibrotic drug. Research has shown that ligustrazine significantly alleviates the occurrence of liver fibrosis by regulating the miR-145 and TGF-β/Smad signalling pathways in BA mouse model (Qiu et al., 2022). Prifenidone and/or andrographolide inhibited the activation of HSCs by blocking the TGF-β/Smad signaling pathway, and the combined treatment synergistically inhibited the phosphorylation of Smad2 and Smad3 in bile duct ligation induced BA mouse model (Xu et al., 2024). This mechanism provides new ideas for clinical applications, especially in diseases such as BA, which can lead to liver fibrosis. Furthermore, antioxidants such as glutathione (GSH) have been shown to improve liver damage caused by BA in experimental models, promoting hepatocyte survival and regeneration (Fried et al., 2020). However, its development in BA faces notable challenges, including the significant incidence of pruritus observed in adult trials for primary biliary cholangitis, which could be particularly problematic in infants already burdened by cholestatic itching (Kanda et al., 2025).

#### 6.3.1 Odevixibat (ileal bile acid transporter inhibitor)

Odevixibat is a highly selective inhibitor of the ileal bile acid transporter (IBAT) that reduces the enterohepatic circulation of bile acids, thereby lowering intrahepatic bile acid concentrations and mitigating cholestatic injury (Levien and Baker, 2023). The pivotal phase III BOLD trial (NCT04336722) is a randomized, double-blind, placebo-controlled study enrolling approximately 200 BA patients who have undergone KPE (Lee and Seki, 2023). The primary composite endpoint is event-free survival at 48 weeks, defined as avoidance of liver transplantation, death, or clinically evident portal hypertension decompensation (Lee and Seki, 2023). Secondary endpoints include serial measurements of serum fibrosis biomarkers, including MMP-7, serum bile acid profiles, liver biochemistry, and growth parameters (Li Z. et al., 2025). An open-label extension study (NCT05426733) is evaluating long-term safety and sustained efficacy (Baumann et al., 2021). While the trial holds promise, its completion is pending, and several questions remain: whether odevixibat can meaningfully alter fibrosis progression beyond bile acid reduction, whether its effects are durable, and whether it can benefit patients who have already developed significant fibrosis.

#### 6.3.2 Obeticholic acid (farnesoid X receptor agonist)

Obeticholic acid (OCA) is a potent semisynthetic FXR agonist with demonstrated direct anti-fibrotic properties through suppression of HSC activation and collagen deposition (Zeng J. et al., 2023). A phase II/III randomized, double-blind, placebo-controlled trial (NCT06121375) is currently recruiting pediatric BA patients who have achieved successful KPE (total bilirubin <2 mg/dL at ≥3 months post-KPE) (Cavallo et al., 2022). The primary endpoint is time to first occurrence of a composite event (death, liver transplant, PELD ≥ 17, or portal hypertension complications) (Cavallo et al., 2022). Key exclusion criteria include BASM, evidence of portal hypertension, and advanced liver dysfunction (INR ≥ 1.5, platelets <120,000/µL) (Cavallo et al., 2022). OCA is being evaluated in conjunction with established local standard of care, with a 6-week dose-titration phase (starting at 1.5 mg adult equivalent dose, titrating to maximum 5 mg) followed by approximately 24 months of treatment (Cavallo et al., 2022). Notable challenges include the significant incidence of pruritus observed in adult PBC trials (which could be particularly problematic in infants already burdened by cholestatic itching), the theoretical risk of hepatotoxicity requiring careful dose titration and monitoring, and the fact that initial safety data in BA patients have not yet been reported (Kanda et al., 2025).

#### 6.3.3 Eicosapentaenoic acid (EPA)

Recent prospective clinical data have demonstrated that eicosapentaenoic acid (EPA), an n-3 polyunsaturated fatty acid, attenuates liver fibrosis progression in BA patients who achieved jaundice clearance post-KPE (Lam et al., 2021). In a study of 57 BA patients, EPA administration (20–40 mg/kg/day) did not improve the rate of jaundice clearance but significantly reduced M2BPGi levels (a validated liver fibrosis marker) at both 1 and 2 years post-KPE, and significantly reduced APRI scores at 2 years (Lam et al., 2021). The mechanism is thought to involve modulation of the n-3/n-6 PUFA ratio, reducing inflammation and oxidative stress (Lam et al., 2021). EPA represents a promising, well-tolerated antifibrotic adjunct that could be integrated into post-KPE management, although larger randomized controlled trials are needed to confirm these findings.

#### 6.3.4 N-acetylcysteine (NAC)

Based on the rationale that NAC provides cysteine for glutathione synthesis-a choleretic and antioxidant agent-a phase 2 trial (NCT03499249) evaluated intravenous NAC (150 mg/kg/day for 7 days) post-KPE (Rajagopalan et al., 2020). The primary endpoint was achieving total serum bile acids ≤10 μmol/L within 24 weeks of KPE. The trial used a two-stage “minimax” design, but the primary endpoint was not met, and no participants were enrolled in stage 2 (Rajagopalan et al., 2020). NAC was generally well-tolerated, with adverse events attributed to the natural clinical course of BA rather than the drug (Rajagopalan et al., 2020). The authors noted that the stringent TSBA cutoff (≤10 μmol/L) may have been too ambitious, as subsequent studies showed that even infants with good bile flow often have TSBA levels up to 322 μmol/L (Rajagopalan et al., 2020). Future trials with less stringent endpoints or longer treatment durations may be warranted.

#### 6.3.5 Mitomycin-C

A prospective randomized controlled trial evaluated local instillation of the antifibrotic agent mitomycin-C (MMC) at the porta hepatis after KPE (Sandberg et al., 2021). The MMC-treated group showed clinically higher native liver survival at 6 months (91% vs. 63%), 1 year (81% vs. 50%), and 2 years (73% vs. 38%) compared to controls, although these differences were not statistically significant (Sandberg et al., 2021). The procedure was technically feasible and associated with no complications (Sandberg et al., 2021). While these results are preliminary, they suggest that local delivery of antifibrotic agents to the porta hepatis represents a novel and potentially effective strategy that merits further investigation in larger trials. Furthermore, we have added a summary (Table 2) for ongoing and completed clinical studies, including the trial phases and key results.

**TABLE 2:** Ongoing and completed clinical studies for BA-associated liver fibrosis.

Agent/intervention	Mechanism of action	Trial phase	Patient population	Key endpoints	Key outcomes/results	Clinical trial identifier/references
Odevixibat	Ileal bile acid transporter (IBAT) inhibitor; reduces enterohepatic bile acid circulation, lowering intrahepatic bile acid concentrations	Phase III (ongoing)	BA patients post-Kasai portoenterostomy (KPE)	Primary: composite event-free survival at 48 weeks (avoidance of LT, death, or clinically evident portal hypertension decompensation); Secondary: MMP-7, serum bile acids, liver biochemistry, growth	Pending completion; open-label extension evaluating long-term safety	NCT04336722; NCT05426733 (Kong and Xiang, 2021; Lee and Seki, 2023; Levien and Baker, 2023; Li Z. et al. 2025)
Obeticholic Acid (OCA)	Farnesoid X receptor (FXR) agonist; suppresses HSC activation, collagen deposition, and has direct anti-fibrotic properties	Phase II/III(ongoing)	Pediatric BA patients with successful KPE (TB < 2 mg/dL at ≥3 months post-KPE); excludes portal hypertension or advanced dysfunction	Primary: time to first composite event (death, LT, PELD ≥17, or portal hypertension complications)	Pending; dose titration (1.5–5 mg adult equivalent) over 6 weeks, then ∼24 months treatment	NCT06121375 (Cavallo et al., 2022; Zeng J. et al., 2023; Kanda et al., 2025)
Eicosapentaenoic Acid (EPA)	n-3 polyunsaturated fatty acid; modulates n−3/n−6 ratio, reduces inflammation and oxidative stress	Prospective observational/pilot	BA patients who achieved jaundice clearance post-KPE	M2BPGi (fibrosis marker), APRI scores at 1 and 2 years	No improvement in jaundice clearance rate; Significant reduction in M2BPGi and APRI at 1 and 2 years post-KPE	Lam et al. (2021)
N-Acetylcysteine (NAC)	Antioxidant; provides cysteine for glutathione synthesis (choleretic and antioxidant effects)	Phase II (completed)	BA patients post-KPE	Primary: total serum bile acids (TSBA) ≤10 μmol/L within 24 weeks of KPE	Primary endpoint not met; no participants enrolled in stage 2; Well-tolerated; AEs attributed to natural disease course rather than drug	NCT03499249 (Rajagopalan et al., 2020)
Mitomycin-C (MMC)	Local antifibrotic agent; inhibits fibroblast proliferation via alkylation	Phase II/pilot RCT	BA patients undergoing KPE	Native liver survival at 6 months, 1 year, and 2 years	Clinically higher native liver survival; Feasible, no complications	(Sandberg et al., 2021) (single-center pilot)
High-dose Corticosteroids (prednisolone)	Immunosuppressive; broad anti-inflammatory	Phase III (completed – START trial)	BA patients post-KPE	Bile drainage at 6 months; native liver survival	No significant benefit in bile drainage at 6 months; Associated with earlier serious adverse events	(Chen et al., 2025) (START trial)
Ursodeoxycholic Acid (UDCA)	Choleretic; enhances bile flow, reduces bile acid toxicity, mild cytoprotective	Observational/standard of care	BA patients post-KPE	Native liver survival, jaundice clearance, liver biochemistry	Mixed evidence; cohort study (n = 141) found UDCA not associated with improved outcomes and linked to hepatic/extrahepatic complications	Dong et al. (2022)

### 6.4 Current controversies and unresolved questions in BA-associated liver fibrosis

Despite the identification of multiple therapeutic targets, the clinical management of progressive fibrosis in BA remains a formidable challenge. This therapeutic bottleneck is largely attributable to our incomplete understanding of the fundamental mechanisms. Therefore, a critical appraisal of the lingering controversies is essential to refine our therapeutic strategies.

The role of viral infection as a primary initiating trigger remains unproven, as human epidemiological studies have failed to identify a consistent viral agent, and most infected infants do not develop BA, suggesting that viral exposure is insufficient alone. Similarly, while immune dysregulation-characterized by CD8^+^ T-cell infiltration, Treg dysfunction, and Th1/Th2 imbalance-is well-documented, the absence of a disease-specific autoantigen and the lack of consistent benefit from immunosuppressive therapies in clinical practice indicate that autoimmunity may be a reactive epiphenomenon rather than a primary driver (Mack et al., 2004). The relative contributions of PFs versus HSCs to the total fibrotic burden are unknown, as current assertions about PF dominance are based on anatomical proximity and *in vitro* sensitivity rather than on quantitative lineage-tracing or cell-specific ablation studies (Zagory et al., 2015). Furthermore, the role of EMT in fibrogenesis is controversial: lineage-tracing studies in mouse models have failed to demonstrate significant transdifferentiation of biliary epithelial cells into collagen-producing MFs, and the phenotypic changes observed may reflect injury-induced plasticity without functional fibrogenic capacity (Omenetti et al., 2011b). Beyond HSCs and PFs, other potential myofibroblast sources-including bone marrow-derived fibrocytes, pericytes, and hepatocytes-remain unexplored in BA.

Additional unresolved questions concern the mechanistic hierarchy between bile acid toxicity and inflammation as drivers of fibrosis progression, particularly in the subset of patients who continue to progress despite successful KPE and improved bile drainage. The reversibility of established fibrosis in BA is another critical knowledge gap; unlike adult chronic liver diseases where fibrosis regresses upon removal of the underlying insult, BA patients often progress to cirrhosis even with adequate bile flow, raising questions about ongoing low-grade injury, irreversible architectural remodeling, or intrinsic limitations of the infant liver to remodel scars. These controversies highlight high-priority research directions, including definitive identification of initiating triggers through unbiased metagenomic and prospective cohort studies, cell-type-specific ablation and lineage-tracing in chronic BA models to quantify myofibroblast origins, functional validation of EMT using conditional gene editing, longitudinal correlation of bile acid profiles and immune signatures with fibrosis trajectories, and investigation of fibrosis regression potential using advanced imaging and biopsy-based metrics. Addressing these knowledge gaps will require multidisciplinary collaboration, standardized biobanking, and the development of more faithful animal models that recapitulate the chronic, progressive fibrosis seen in human BA, ultimately enabling better patient stratification and the design of rationally targeted combination therapies.

### 6.5 Single-cell transcriptomic insights into BA-associated liver fibrosis

Many of the persistent controversies in BA-such as the relative contribution of PFs versus HSCs, or the role of EMT-stem largely from the limitations of traditional bulk RNA sequencing and immunohistochemistry, which cannot resolve the complex cellular heterogeneity within the fibrotic niche. The advent of single-cell sequencing technology has recently provided a powerful lens to dissect these intricate interactions at unprecedented resolution.

Single-cell RNA sequencing (scRNA-seq) and integrated spatial transcriptomics have fundamentally reshaped our understanding of liver fibrosis in BA, moving beyond the traditional view of uniform cellular responses to reveal remarkable heterogeneity across HSCs, biliary epithelial cells (BECs), and immune populations (Shen et al., 2019; Kong et al., 2026; Blakeley et al., 2026). In BA-induced fibrosis, HSCs do not simply transition from quiescence to activation as a single population; instead, they differentiate into multiple transcriptionally distinct subtypes, including classical profibrotic subtypes marked by high COL1A1 expression, inflammatory-responsive subtypes enriched for cytokine signaling, and proliferative precursor-like subtypes with enhanced cell cycle genes (Li et al., 2026). Pseudotime trajectory analyses further delineate a differentiation continuum from quiescence through intermediate states to fully activated MFs, uncovering transitional phases that may represent therapeutic windows for early intervention. Similarly, BECs exhibit striking phenotypic diversity, with reactive proliferative subpopulations undergoing EMT characterized by upregulated mesenchymal markers, SOX9, and CK19, while secreting chemokines such as CCL2 and CXCL1 that recruit immune cells to portal areas (Li et al., 2026). This epithelial plasticity challenges the paradigm that fibrosis is driven solely by HSCs, implicating BECs as direct contributors to extracellular matrix remodeling through EMT-related pathways including TGF-β and Wnt/β-catenin. The immune microenvironment is equally reshaped, with macrophages displaying marked polarization into pro-fibrotic scar-associated macrophages (SAMs) expressing CD163 and SPP1, which secrete TGF-β and osteopontin to activate HSCs (Li et al., 2026). Temporal dynamics reveal that intermediate CXCL10+ monocytes dominate early stages, whereas TREM2+ SAMs become prominent in advanced fibrosis, suggesting stage-specific roles. Concurrently, CD8^+^ T cells exhibit exhaustion with upregulated PD-1 and TIM-3, while regulatory T cell populations expand, fostering an immunosuppressive milieu that may facilitate fibrosis progression (Blakeley et al., 2026). Collectively, these single-cell insights establish a refined cellular framework wherein HSC heterogeneity, BEC plasticity, and immune remodeling interact within a complex fibrotic niche, offering multiple discrete cellular targets for antifibrotic strategies tailored to BA.

At the molecular level, single-cell analyses have uncovered cell-type-specific activation of key signaling pathways rather than uniform global responses, with TGF-β signaling showing elevated SMAD2/3 phosphorylation specifically in HSCs and MFs, while hepatocytes exhibit suppressed activity, indicating a protective phenotype (Li Z. et al., 2025). The Hippo/YAP pathway is aberrantly activated in reactive cholangiocytes, promoting proliferation and upregulating fibrosis-associated genes, and synergizes with TGF-β to enhance matrix accumulation within a pathogenic cholangiocyte niche. Weighted gene co-expression network analysis further identifies WNT/β-catenin pathway enrichment in specific myofibroblast subpopulations, sustaining their proliferative and ECM-producing functions (Yang et al., 2025). Ligand-receptor interaction reconstructions reveal a complex intercellular communication network centered on damaged cholangiocytes secreting CCL2 and CX3CL1 to recruit monocytes, which differentiate into SAMs and amplify fibrogenesis through a positive feedback loop involving PDGF and FGF signaling with activated HSCs. Spatial transcriptomics confirms that these interactions are concentrated in peribiliary and fibrotic bridging zones, highlighting localized microenvironmental imbalances as key drivers (Ye et al., 2022). Epigenetically, combined single-cell ATAC-seq and RNA-seq analyses demonstrate that profibrotic gene loci in HSCs and macrophages exhibit increased chromatin accessibility, establishing a stable fibrotic memory that persists even after initial injury resolution (Wang J. et al., 2023). Transcription factors such as AP-1 and ETS family members are enriched at accessible regions, and differential histone marks H3K27ac and H3K4me3 across myofibroblast subsets indicate specialized regulatory landscapes governing diverse fibrogenic functions. This integrated molecular framework-encompassing cell-type-specific pathway activation, spatially organized cell-cell communication, and epigenetic memory-provides a mechanistic basis for fibrosis persistence and reveals novel therapeutic nodes, including YAP inhibitors, SPP1 blocking antibodies, and epigenetic modulators targeting chromatin states, which could overcome the limitations of conventional antifibrotic agents that fail to account for cellular heterogeneity.

The translational prospects of single-cell sequencing in BA are substantial yet accompanied by significant challenges that must be addressed for clinical implementation. In diagnostic biomarker discovery, single-cell transcriptomics has identified cell-type-specific markers such as THBS2 and COL10A1 in activated HSCs and CHI3L1 in macrophages, with combinatorial ECM protein panels (including Col4a3, PCOLCE, and LUM) demonstrating superior specificity and sensitivity over conventional serum markers like hyaluronic acid and PIIINP in validation cohorts (Bai et al., 2022; Xiao et al., 2019). However, technical batch effects, limited sample sizes, high costs, and the invasive nature of liver biopsies impede large-scale validation and routine application, necessitating multicenter studies, standardized protocols, and emerging minimally invasive liquid biopsy approaches. For therapeutic target identification, single-cell profiling has revealed why agents like Cenicriviroc show limited efficacy-they target only CCR2+ macrophage subsets while other pro-fibrotic populations remain unresponsive-and has instead pinpointed novel candidates such as VGLL3 in the Hippo/YAP pathway and SPP1 signaling in macrophages, with single-cell CRISPR screening accelerating functional validation (Xue et al., 2024; Shashni et al., 2023; Pan et al., 2026). Nevertheless, the complexity of compensatory mechanisms and cell-cell interactions likely demands combination therapies, and ensuring drug delivery specificity to fibrogenic subsets while minimizing systemic toxicity remains a critical hurdle. Personalized treatment frameworks, enabled by molecular subtyping of BA patients into immune-dominant, myofibroblast-dominant, or mixed phenotypes, promise to guide tailored interventions-immunomodulatory therapies for immune-dominant cases, HSC-targeted agents for myofibroblast-dominant patients, or combination regimens for mixed types-and may predict responses to KPE based on preoperative immune and HSC activation states (Zhang and Zhang, 2025). Yet widespread adoption is constrained by cost, technical complexity, and the need for sophisticated bioinformatics expertise. Overcoming these barriers through integrative multi-omics approaches, longitudinal cohort studies, and user-friendly analytical tools will be essential to translate single-cell insights into clinical practice. Ultimately, while challenges remain, single-cell sequencing holds transformative potential to shift BA management toward precise, personalized strategies that improve prognostication, therapeutic efficacy, and long-term outcomes for affected children.

## 7 Future perspectives and conclusions

BA-associated liver fibrosis represents a paradigm of complex, multifactorial disease progression wherein genetic predisposition, developmental anomalies, immune dysregulation, and metabolic injury converge to drive relentless extracellular matrix deposition and architectural destruction. This review has systematically delineated the hierarchical genetic architecture-from ciliopathy-related variants (PKD1L1) and developmental susceptibility genes (ADD3, GPC1) to immune-modulating polymorphisms and pro-fibrotic effectors-that collectively establish a primed fibrotic milieu. We have elucidated the critical contributions of aberrantly activated cellular players, including biliary epithelial cells undergoing EMT, transdifferentiated hepatic stellate cells and PFs, and immune cells polarizing toward pro-fibrotic phenotypes, all orchestrated through the integrated signaling network of TGF-β/Smad, Wnt/β-catenin, Hippo/YAP, and Notch pathways, with epigenetic modifications providing a sustaining memory of fibrogenic activation (Kong et al., 2025a; Kong et al., 2025b). Diagnostic modalities have advanced substantially, with shear wave elastography and serum matrix metalloproteinase-7 offering non-invasive risk stratification, yet their clinical integration remains hampered by standardization gaps and accessibility disparities. Therapeutically, while KPE continues as the cornerstone intervention, its inherent limitations and the failure of adjunctive agents to demonstrate consistent benefit underscore the urgent need for mechanism-based pharmacotherapies; ongoing trials of odevixibat and obeticholic acid represent cautiously optimistic developments, but their long-term efficacy and safety in infants require rigorous validation.

Critically, several unresolved controversies impede translational progress. The relative primacy of viral triggers versus intrinsic developmental defects, the functional contribution of EMT versus portal fibroblast activation to fibrotic burden, and the mechanistic hierarchy between bile acid toxicity and immune-driven injury remain inadequately defined. The absence of BA-specific fibrosis staging systems, the over-reliance on acute murine models that fail to recapitulate chronic progressive human disease, and the limited understanding of fibrosis reversibility in the infant liver collectively constrain therapeutic innovation. However, the advent of single-cell and spatial transcriptomic technologies is revolutionizing our comprehension of cellular heterogeneity, revealing transcriptionally distinct hepatic stellate cell subpopulations, reactive biliary epithelial cell states, and scar-associated macrophage phenotypes that may represent discrete therapeutic nodes. These high-resolution approaches, coupled with patient-derived organoid platforms and multi-omics integration, offer unprecedented opportunities for molecular subtyping and precision medicine.

Looking forward, we propose a strategic roadmap for the next decade of BA fibrosis research: (i) establish large-scale, international, multi-omic consortia to comprehensively characterize human BA livers at single-cell resolution, validating murine-derived hypotheses and identifying conserved disease-driving mechanisms; (ii) develop and deploy human-relevant *ex vivo* platforms-including cholangiocyte organoids and liver-on-a-chip systems-for personalized drug screening and mechanistic interrogation; (iii) implement molecular subtyping based on genetic, epigenetic, and transcriptomic signatures to stratify patients for targeted combination therapies addressing both developmental anomalies and fibrotic cascades; (iv) accelerate the development of low-cost, portable ultrasound devices integrated with artificial intelligence-driven automated interpretation for global deployment, particularly in resource-limited regions where BA incidence is disproportionately high; (v) conduct rigorously designed, biomarker-enriched clinical trials with endpoints that reflect meaningful fibrosis modification rather than surrogate biochemical improvements; and (vi) foster multidisciplinary collaboration bridging basic scientists, clinicians, bioinformaticians, and public health experts to ensure equitable access to diagnostic and therapeutic innovations.

Ultimately, conquering BA-associated liver fibrosis demands a fundamental paradigm shift-from acute, reductionist models toward chronic, human-centric systems; from one-size-fits-all approaches toward molecularly guided precision medicine; and from technology-centric solutions toward globally accessible, equitable care. By embracing this integrated vision and sustaining collaborative commitment, we can substantially improve native liver survival, reduce the burden of liver transplantation, and transform the long-term prognosis for children afflicted with this devastating disease. The path forward is challenging, but the convergence of technological innovation, mechanistic insight, and clinical determination renders this goal increasingly attainable.

## References

[B1] Adeyemi A. States L. Wann L. Lin H. C. Rand E. B. (2020). Biliary excretion noted on hepatobiliary iminodiacetic acid scan does not exclude diagnosis of biliary atresia. J. Pediatr. 220, 245–248. 10.1016/j.jpeds.2019.12.055 32111380

[B2] Allam A. El-Guindi M. Konsowa H. El Azab D. Allam M. Salem T. (2019). Expression of vascular endothelial growth factor A in liver tissues of infants with biliary atresia. Clin. Exp. Hepatol. 5, 308–316. 10.5114/ceh.2019.89476 31893243 PMC6935852

[B3] Anouti A. Patel M. S. VanWagner L. B. Lee W. M. Fung J. J. Cholankeril G. (2023). Biliary atresia and liver transplantation in the United States: a contemporary analysis. Liver Int. 43, 2198–2209. 10.1111/liv.15689 37548078

[B4] Åsberg A. Løfblad L. Hov G. G. Mikkelsen G. (2023). Reference change values of FIB-4. Scand. J. Clin. Lab. Invest. 83, 394–396. 10.1080/00365513.2023.2241363 37504797

[B5] Aseem S. O. Huebert R. C. (2019). Epigenetic mechanisms of pancreatobiliary fibrosis. Curr. Treat. Options Gastroenterol. 17, 342–356. 10.1007/s11938-019-00239-0 31300946 PMC6687535

[B6] Ayabe H. DePasquale E. A. K. Amarachintha S. P. Mourya R. Li W. Nalluri S. (2025). Cellular crosstalk mediated by TGF-β drives epithelial-mesenchymal transition in patient-derived multi-compartment biliary organoids. Nat. Commun. 16, 6575. 10.1038/s41467-025-61442-5 40675983 PMC12271497

[B7] Bai M. R. Niu W. B. Zhou Y. Gong Y. M. Lu Y. J. Yu X. X. (2020). Association of common variation in ADD3 and GPC1 with biliary atresia susceptibility. Aging (Albany NY) 12, 7163–7182. 10.18632/aging.103067 32315284 PMC7202506

[B8] Bai Y. Wang P. Qi Y. Li Y. Liu W. Gao L. (2022). Diagnostic value of HA, PC-III, IV-C, and LN in infants with congenital biliary atresia. Med. (Baltimore) 101 (32), e29752. 10.1097/MD.0000000000029752 35960122 PMC9371546

[B9] Bakalenko N. Kuznetsova E. Malashicheva A. (2024). The complex interplay of TGF-β and Notch signaling in the pathogenesis of fibrosis. Int. J. Mol. Sci. 25, 10803. 10.3390/ijms251910803 39409132 PMC11477142

[B10] Bansal R. van Baarlen J. Storm G. Prakash J. (2015). The interplay of the Notch signaling in hepatic stellate cells and macrophages determines the fate of liver fibrogenesis. Sci. Rep. 5, 18272. 10.1038/srep18272 26658360 PMC4677309

[B11] Barcena-Varela M. Colyn L. Fernandez-Barrena M. G. (2019). Epigenetic mechanisms in hepatic stellate cell activation during liver fibrosis and carcinogenesis. Int. J. Mol. Sci. 20, 2507. 10.3390/ijms20102507 31117267 PMC6566358

[B12] Basta M. D. Petruk S. Mazo A. Walker J. L. (2023). Fibrosis-the tale of H3K27 histone methyltransferases and demethylases. Front. Cell Dev. Biol. 11, 1193344. 10.3389/fcell.2023.1193344 37476157 PMC10354294

[B13] Bates J. Vijayakumar A. Ghoshal S. Marchand B. Yi S. Kornyeyev D. (2020). Acetyl-CoA carboxylase inhibition disrupts metabolic reprogramming during hepatic stellate cell activation. J. Hepatol. 73, 896–905. 10.1016/j.jhep.2020.04.037 32376414

[B14] Baumann U. Sturm E. Lacaille F. Gonzalès E. Arnell H. Fischler B. (2021). Effects of odevixibat on pruritus and bile acids in children with cholestatic liver disease: phase 2 study. Clin. Res. Hepatol. Gastroenterol. 45, 101751. 10.1016/j.clinre.2021.101751 34182185

[B15] Beckmann S. Simanowski J. H. (2020). Update in contrast-enhanced ultrasound. Visc. Med. 36, 476–486. 10.1159/000511352 33447604 PMC7768106

[B16] Berauer J. P. Mezina A. I. Okou D. T. Sabo A. Muzny D. M. Gibbs R. A. (2019). Identification of polycystic kidney disease 1 like 1 gene variants in children with biliary atresia splenic malformation syndrome. Hepatology 70, 899–910. 10.1002/hep.30515 30664273 PMC6642859

[B17] Bessho K. Bezerra J. A. (2011). Biliary atresia: will blocking inflammation tame the disease? Annu. Rev. Med. 62, 171–185. 10.1146/annurev-med-042909-093734 21226614 PMC4096311

[B18] Blakeley P. D. Liu F. Wu Z. Tang C. S. M. Tam P. K. H. Wong K. K. H. (2026). Single-cell transcriptomics reveals anti-inflammatory effects of prednisolone on the hepatic niche in biliary atresia. Sci. Rep. 10.1038/s41598-026-48897-2 PMC13469716 42156429

[B19] Cavallo L. Kovar E. M. Aqul A. McLoughlin L. Mittal N. K. Rodriguez-Baez N. (2022). The epidemiology of biliary atresia: exploring the role of developmental factors on birth prevalence. J. Pediatr. 246, 89–94.e82. 10.1016/j.jpeds.2022.03.038 35364097 PMC9332904

[B20] Ceci L. Gaudio E. Kennedy L. (2024). Cellular interactions and crosstalk facilitating biliary fibrosis in cholestasis. Cell Mol. Gastroenterol. Hepatol. 17, 553–565. 10.1016/j.jcmgh.2024.01.005 38216052 PMC10883986

[B21] Chandrashekaran V. Das S. Seth R. K. Dattaroy D. Alhasson F. Michelotti G. (2016). Purinergic receptor X7 mediates leptin induced GLUT4 function in stellate cells in nonalcoholic steatohepatitis. Biochim. Biophys. Acta 1862, 32–45. 10.1016/j.bbadis.2015.10.009 26474534 PMC4988689

[B22] Chen J. K. (2016). I only have eye for ewe: the discovery of cyclopamine and development of Hedgehog pathway-targeting drugs. Nat. Prod. Rep. 33, 595–601. 10.1039/c5np00153f 26787175 PMC4856577

[B23] Chen Y. Choi S. S. Michelotti G. A. Chan I. S. Swiderska-Syn M. Karaca G. F. (2012). Hedgehog controls hepatic stellate cell fate by regulating metabolism. Gastroenterology 143, 1319–1329.e1311. 10.1053/j.gastro.2012.07.115 22885334 PMC3480563

[B24] Chen J. Jiang X. Chen Y. Tang H. Zhang Y. Lu Y. (2025). Clinical significance of fibrosis 4 index in early-stage hepatocellular carcinoma patients received ultrasound-guided microwave ablation. Appl. Biochem. Biotechnol. 197, 1650–1661. 10.1007/s12010-024-05108-w 39601976

[B25] Cheng Y. He C. Y. Tian D. Y. Chen S. H. Ren J. R. Sun H. L. (2023). Physiological β-amyloid clearance by the liver and its therapeutic potential for Alzheimer's disease. Acta Neuropathol. 145, 717–731. 10.1007/s00401-023-02559-z 36964213

[B26] Cofer Z. C. Cui S. EauClaire S. F. Kim C. Tobias J. W. Hakonarson H. (2016). Methylation microarray studies highlight PDGFA expression as a factor in biliary atresia. PLoS One 11, e0151521. 10.1371/journal.pone.0151521 27010479 PMC4806872

[B27] de La Taille E. Sales de Gauzy J. Gaubert Noirot M. (2021). Idiopathic clubfoot treatment and heterogeneity of current therapeutic strategies: the Ponseti method versus the French functional method (a systematic review). Arch. Pediatr. 28, 422–428. 10.1016/j.arcped.2021.04.003 34020862

[B28] de Lau W. Peng W. C. Gros P. Clevers H. (2014). The R-spondin/Lgr5/Rnf43 module: regulator of Wnt signal strength. Genes Dev. 28, 305–316. 10.1101/gad.235473.113 24532711 PMC3937510

[B29] Dong B. Weng Z. Lyu G. Yang X. Wang H. (2022). The diagnostic performance of ultrasound elastography for biliary atresia: a meta-analysis. Front. Public Health 10, 973125. 10.3389/fpubh.2022.973125 36388297 PMC9643747

[B30] Donnelly J. M. Engevik A. C. Engevik M. Schumacher M. A. Xiao C. Yang L. (2014). Gastritis promotes an activated bone marrow-derived mesenchymal stem cell with a phenotype reminiscent of a cancer-promoting cell. Dig. Dis. Sci. 59, 569–582. 10.1007/s10620-013-2927-z 24202649 PMC4301577

[B31] Dranoff J. A. Wells R. G. (2010). Portal fibroblasts: underappreciated mediators of biliary fibrosis. Hepatology 51, 1438–1444. 10.1002/hep.23405 20209607 PMC2850946

[B32] Du K. Hyun J. Premont R. T. Choi S. S. Michelotti G. A. Swiderska-Syn M. (2018). Hedgehog-YAP signaling pathway regulates glutaminolysis to control activation of hepatic stellate cells. Gastroenterology 154, 1465–1479.e1413. 10.1053/j.gastro.2017.12.022 29305935 PMC5880682

[B33] Du K. Chitneni S. K. Suzuki A. Wang Y. Henao R. Hyun J. (2020). Increased glutaminolysis marks active scarring in nonalcoholic steatohepatitis progression. Cell Mol. Gastroenterol. Hepatol. 10, 1–21. 10.1016/j.jcmgh.2019.12.006 31881361 PMC7215180

[B34] Edom P. T. Meurer L. da Silveira T. R. Matte U. dos Santos J. L. (2011). Immunolocalization of VEGF A and its receptors, VEGFR1 and VEGFR2, in the liver from patients with biliary atresia. Appl. Immunohistochem. Mol. Morphol. 19, 360–368. 10.1097/PAI.0b013e3182028a8e 21285868

[B35] Fabris L. Brivio S. Cadamuro M. Strazzabosco M. (2016). Revisiting epithelial-to-mesenchymal transition in liver fibrosis: clues for a better understanding of the “reactive” biliary epithelial phenotype. Stem Cells Int. 2016, 2953727. 10.1155/2016/2953727 26880950 PMC4736590

[B36] Fabris L. Spirli C. Cadamuro M. Fiorotto R. Strazzabosco M. (2017). Emerging concepts in biliary repair and fibrosis. Am. J. Physiol. Gastrointest. Liver Physiol. 313, G102–g116. 10.1152/ajpgi.00452.2016 28526690 PMC5582882

[B37] Fabris L. Fiorotto R. Spirli C. Cadamuro M. Mariotti V. Perugorria M. J. (2019). Pathobiology of inherited biliary diseases: a roadmap to understand acquired liver diseases. Nat. Rev. Gastroenterol. Hepatol. 16, 497–511. 10.1038/s41575-019-0156-4 31165788 PMC6661007

[B38] Ferraioli G. Wong V. W. S. Castera L. Berzigotti A. Sporea I. Dietrich C. F. (2018). Liver ultrasound elastography: an update to the world federation for ultrasound in medicine and biology guidelines and recommendations. Ultrasound Med. Biol. 44, 2419–2440. 10.1016/j.ultrasmedbio.2018.07.008 30209008

[B39] Fligor S. C. Hirsch T. I. Tsikis S. T. Adeola A. Puder M. (2022). Current and emerging adjuvant therapies in biliary atresia. Front. Pediatr. 10, 1007813. 10.3389/fped.2022.1007813 36313875 PMC9614654

[B40] Frassetto R. Parolini F. Marceddu S. Satta G. Papacciuoli V. Pinna M. A. (2018). Intrahepatic bile duct primary cilia in biliary atresia. Hepatol. Res. 48, 664–674. 10.1111/hepr.13060 29330965

[B41] Fried S. Gilboa D. Har-Zahav A. Lavrut P. M. Du Y. Karjoo S. (2020). Extrahepatic cholangiocyte obstruction is mediated by decreased glutathione, Wnt and Notch signaling pathways in a toxic model of biliary atresia. Sci. Rep. 10, 7599. 10.1038/s41598-020-64503-5 32371929 PMC7200694

[B42] Fried S. Har-Zahav A. Hamudi Y. Mahameed S. Mansur R. Dotan M. (2025). Biliary atresia: insights into mechanisms using a toxic model of the disease including Wnt and Hippo signaling pathways and microtubules. Pediatr. Res. 97, 184–194. 10.1038/s41390-024-03335-9 38914763 PMC11798875

[B43] Fuji H. Miller G. Nishio T. Koyama Y. Lam K. Zhang V. (2021). The role of Mesothelin signaling in portal fibroblasts in the pathogenesis of cholestatic liver fibrosis. Front. Mol. Biosci. 8, 790032. 10.3389/fmolb.2021.790032 34966784 PMC8710774

[B44] Gajendiran P. Vega L. I. Itoh K. Sesaki H. Vakili M. R. Lavasanifar A. (2018). Elevated mitochondrial activity distinguishes fibrogenic hepatic stellate cells and sensitizes for selective inhibition by mitotropic doxorubicin. J. Cell Mol. Med. 22, 2210–2219. 10.1111/jcmm.13501 29397578 PMC5867155

[B45] Gao Y. Zhu L. Xiao H. Yang C. Xu J. Mou F. (2023). Clinical study of the value of shear wave elastography in evaluating the degree of liver fibrosis in children. Abdom. Radiol. (NY) 48, 1298–1305. 10.1007/s00261-023-03837-w 36811726

[B46] Garcia-Barceló M. M. Yeung M. Y. Miao X. P. Tang C. S. M. Cheng G. Chen G. (2010). Genome-wide association study identifies a susceptibility locus for biliary atresia on 10q24.2. Hum. Mol. Genet. 19, 2917–2925. 10.1093/hmg/ddq196 20460270 PMC2893814

[B47] Geisler F. Strazzabosco M. (2015). Emerging roles of Notch signaling in liver disease. Hepatology 61, 382–392. 10.1002/hep.27268 24930574 PMC4268103

[B48] Gupta K. Xu J. P. Diamond T. de Jong I. E. M. Glass A. Llewellyn J. (2024). Low-dose biliatresone treatment of pregnant mice causes subclinical biliary disease in their offspring: evidence for a spectrum of neonatal injury. PLoS One 19, e0301824. 10.1371/journal.pone.0301824 38578745 PMC10997102

[B49] Gupta K. Llewellyn J. Roberts E. Liu C. Naji A. Assoian R. K. (2025). Biliary atresia susceptibility gene EFEMP1 regulates extrahepatic bile duct elastic fiber formation and mechanics. JHEP Rep. 7, 101215. 10.1016/j.jhepr.2024.101215 39717503 PMC11663959

[B50] Hellen D. J. Bennett A. Malla S. Klindt C. Rao A. Dawson P. A. (2023). Liver-restricted deletion of the biliary atresia candidate gene Pkd1l1 causes bile duct dysmorphogenesis and ciliopathy. Hepatology 77, 1274–1286. 10.1097/hep.0000000000000029 36645229 PMC12440248

[B51] Hellerbrand C. Stefanovic B. Giordano F. Burchardt E. R. Brenner D. A. (1999). The role of TGFbeta1 in initiating hepatic stellate cell activation *in vivo* . J. Hepatol. 30, 77–87. 10.1016/s0168-8278(99)80010-5 9927153

[B52] Horn P. Tacke F. (2024). Metabolic reprogramming in liver fibrosis. Cell Metab. 36, 1439–1455. 10.1016/j.cmet.2024.05.003 38823393

[B53] Iordanskaia T. Hubal M. J. Koeck E. Rossi C. Schwarz K. Nadler E. P. (2013). Dysregulation of upstream and downstream transforming growth factor-β transcripts in livers of children with biliary atresia and fibrogenic gene signatures. J. Pediatr. Surg. 48, 2047–2053. 10.1016/j.jpedsurg.2013.03.047 24094956 PMC3792400

[B54] Jain M. Rivera S. Monclus E. A. Synenki L. Zirk A. Eisenbart J. (2013). Mitochondrial reactive oxygen species regulate transforming growth factor-β signaling. J. Biol. Chem. 288, 770–777. 10.1074/jbc.M112.431973 23204521 PMC3543026

[B55] Jang S. Choi N. Park J. H. Yoo K. H. (2025). Contribution of histone deacetylases (HDACs) to the regulation of histone and non-histone proteins: implications for fibrotic diseases. BMB Rep. 58, 313–324. 10.5483/BMBRep.2024-0122 40635201 PMC12402695

[B56] Jiang Y. Xiang C. Zhong F. Zhang Y. Wang L. Zhao Y. (2021). Histone H3K27 methyltransferase EZH2 and demethylase JMJD3 regulate hepatic stellate cells activation and liver fibrosis. Theranostics 11, 361–378. 10.7150/thno.46360 33391480 PMC7681085

[B57] Jiang J. Dong R. Du M. Chen G. Yang J. Xie X. (2024). Serum matrix metalloproteinase-7 for discriminating biliary atresia: a diagnostic accuracy and validation study. J. Transl. Med. 22, 636. 10.1186/s12967-024-05442-x 38978022 PMC11229253

[B58] Jiang X. Zhao W. Shen B. Han Y. Chen K. (2025). CD47-mediated regulation of glucose and lipid metabolism: implications for the pathogenesis of MASLD. Front. Endocrinol. (Lausanne) 16, 1535382. 10.3389/fendo.2025.1535382 40630093 PMC12234329

[B59] Jin Y. Li C. Xu D. Zhu J. Wei S. Zhong A. (2020). Jagged1-mediated myeloid Notch1 signaling activates HSF1/Snail and controls NLRP3 inflammasome activation in liver inflammatory injury. Cell Mol. Immunol. 17, 1245–1256. 10.1038/s41423-019-0318-x 31673056 PMC7784844

[B60] Kanda T. Sasaki-Tanaka R. Kimura N. Abe H. Yoshida T. Hayashi K. (2025). Pruritus in chronic cholestatic liver diseases, especially in primary biliary cholangitis: a narrative review. Int. J. Mol. Sci. 26, 1883. 10.3390/ijms26051883 40076514 PMC11900276

[B61] Karin D. Koyama Y. Brenner D. Kisseleva T. (2016). The characteristics of activated portal fibroblasts/myofibroblasts in liver fibrosis. Differentiation 92, 84–92. 10.1016/j.diff.2016.07.001 27591095 PMC5079826

[B62] Karpen S. J. Kelly D. Mack C. Stein P. (2020). Ileal bile acid transporter inhibition as an anticholestatic therapeutic target in biliary atresia and other cholestatic disorders. Hepatol. Int. 14, 677–689. 10.1007/s12072-020-10070-w 32653991

[B63] Ke J. Zeng S. Mao J. Wang J. Lou J. Li J. (2016). Common genetic variants of GPC1 gene reduce risk of biliary atresia in a Chinese population. J. Pediatr. Surg. 51, 1661–1664. 10.1016/j.jpedsurg.2016.05.009 27373597

[B64] Kitto L. J. Henderson N. C. (2021). Hepatic stellate cell regulation of liver regeneration and repair. Hepatol. Commun. 5, 358–370. 10.1002/hep4.1628 33681672 PMC7917274

[B65] Kong M. Xiang B. (2021). Identifying biomarkers to predict the prognosis of biliary atresia by weighted gene co-expression network analysis. Front. Genet. 12, 760182. 10.3389/fgene.2021.760182 34899846 PMC8656673

[B66] Kong M. Zhai Y. Liu H. Zhang S. Chen S. Li W. (2025a). Insights into the mechanisms of angiogenesis in hepatoblastoma. Front. Cell Dev. Biol. 13, 1535339. 10.3389/fcell.2025.1535339 40438141 PMC12116456

[B67] Kong M. Zhang S. T. Ma X. (2025b). Insights into the mechanisms of microRNAs in hepatoblastoma: from diagnosis to treatment. Precis. Clin. Med. 8, pbaf034. 10.1093/pcmedi/pbaf034 41393242 PMC12699220

[B68] Kong M. Jia J. Liu C. Liu H. Zhang S. (2026). Liver fibrosis in biliary atresia: identification of the key gene EDIL3 via integrated bioinformatics. Front. Med. (Lausanne) 12, 1726141. 10.3389/fmed.2025.1726141 41647012 PMC12868289

[B69] Koyama Y. Wang P. Liang S. Iwaisako K. Liu X. Xu J. (2017). Mesothelin/mucin 16 signaling in activated portal fibroblasts regulates cholestatic liver fibrosis. J. Clin. Invest. 127, 1254–1270. 10.1172/jci88845 28287406 PMC5373891

[B70] Kumar S. Duan Q. Wu R. Harris E. N. Su Q. (2021). Pathophysiological communication between hepatocytes and non-parenchymal cells in liver injury from NAFLD to liver fibrosis. Adv. Drug Deliv. Rev. 176, 113869. 10.1016/j.addr.2021.113869 34280515 PMC11792083

[B71] Kurabekova R. M. Gichkun O. E. Tsirulnikova O. M. Pashkova I. E. Fomina V. A. Shevchenko O. P. (2023). Analysis of the association between the Tgfb1 gene haplotype and liver diseases in children. Acta Naturae 15, 75–81. 10.32607/actanaturae.19425 PMC10615185 37908775

[B72] Lally J. S. V. Ghoshal S. DePeralta D. K. Moaven O. Wei L. Masia R. (2019). Inhibition of acetyl-coa carboxylase by phosphorylation or the inhibitor ND-654 suppresses lipogenesis and hepatocellular carcinoma. Cell Metab. 29, 174–182.e175. 10.1016/j.cmet.2018.08.020 30244972 PMC6643297

[B73] Lam W. Y. Tang C. S. M. So M. T. Yue H. Hsu J. S. Chung P. H. Y. (2021). Identification of a wide spectrum of ciliary gene mutations in nonsyndromic biliary atresia patients implicates ciliary dysfunction as a novel disease mechanism. EBioMedicine 71, 103530. 10.1016/j.ebiom.2021.103530 34455394 PMC8403738

[B74] Lee S. J. Kim K. H. Pak S. C. Kang Y. N. Yoon G. S. Park K. K. (2015). Notch signaling affects biliary fibrosis via transcriptional regulation of RBP-jκ in an animal model of chronic liver disease. Int. J. Clin. Exp. Pathol. 8, 12688–12697. 26722458 PMC4680403

[B75] Lee Y. S. Seki E. (2023). *In vivo* and *in vitro* models to study liver fibrosis: mechanisms and limitations. Cell Mol. Gastroenterol. Hepatol. 16, 355–367. 10.1016/j.jcmgh.2023.05.010 37270060 PMC10444957

[B76] Levien T. L. Baker D. E. (2023). Formulary drug review: odevixibat. Hosp. Pharm. 58, 125–133. 10.1177/00185787211069036 36890950 PMC9986570

[B77] Li Z. Li B. Pan J. Jin J. (2014). TNF-α enhances the effect of TGF-β on Gli2 expression in the KG-1 leukemic cell line. Exp. Ther. Med. 8, 676–680. 10.3892/etm.2014.1743 25009639 PMC4079412

[B78] Li K. Zhang X. Yang L. Wang X. X. Yang D. H. Cao G. Q. (2016). Foxp3 promoter methylation impairs suppressive function of regulatory T cells in biliary atresia. Am. J. Physiol. Gastrointest. Liver Physiol. 311, G989–G997. 10.1152/ajpgi.00032.2016 27659419

[B79] Li F. B. Zhao H. Peng K. R. Gao Z. G. Huang S. J. Tou J. F. (2016). Expression of transforming growth factor-β1 and connective tissue growth factor in congenital biliary atresia and neonatal hepatitis liver tissue. Genet. Mol. Res. 15. 10.4238/gmr.15017217 26909983

[B80] Li X. Liu S. Li T. Yang Q. Chen Y. Meng Y. (2025). Inflammatory factor CCL2 enhances the interaction between monocyte-macrophage cells and liver parenchymal cells to promote liver inflammation and fibrosis in biliary atresia. BMC Pediatr. 25 (1), 643. 10.1186/s12887-025-05984-z 40849451 PMC12374366

[B81] Li Z. Deng Y. Liao Q. (2025). The diagnostic accuracy of MMP-7 for the diagnosis for biliary atresia-a systematic review and meta-analysis. BMC Pediatr. 25, 652. 10.1186/s12887-025-06032-6 40855281 PMC12376742

[B82] Li X. Li T. Liu S. Zhao Y. Chen Y. Abudureyimu A. (2026). Scar-associated macrophages and biliary epithelial cells interaction exacerbates hepatic fibrosis in biliary atresia. Pediatr. Res. 99, 362–374. 10.1038/s41390-025-04100-2 40383871

[B83] Lim Y. Z. Zhu M. Wang Y. Sharma T. Kelley S. Oertling E. (2024). Pkd1l1-deficiency drives biliary atresia through ciliary dysfunction in biliary epithelial cells. J. Hepatol. 81, 62–75. 10.1016/j.jhep.2024.02.031 38460793

[B84] Lin J. Chen A. (2011). Curcumin diminishes the impacts of hyperglycemia on the activation of hepatic stellate cells by suppressing membrane translocation and gene expression of glucose transporter-2. Mol. Cell Endocrinol. 333, 160–171. 10.1016/j.mce.2010.12.028 21195127 PMC3039105

[B85] Lin Z. Xie X. Lin H. Fu M. Su L. Tong Y. (2018). Epistatic association of CD14 and NOTCH2 genetic polymorphisms with biliary atresia in a southern chinese population. Mol. Ther. Nucleic Acids 13, 590–595. 10.1016/j.omtn.2018.10.006 30439647 PMC6234514

[B86] Lisboa F. A. Warren J. Sulkowski G. Aparicio M. David G. Zudaire E. (2011). Pregnancy-specific glycoprotein 1 induces endothelial tubulogenesis through interaction with cell surface proteoglycans. J. Biol. Chem. 286, 7577–7586. 10.1074/jbc.M110.161810 21193412 PMC3045012

[B87] Liu R. M. Desai L. P. (2015). Reciprocal regulation of TGF-β and reactive oxygen species: a perverse cycle for fibrosis. Redox Biol. 6, 565–577. 10.1016/j.redox.2015.09.009 26496488 PMC4625010

[B88] Liu Z. Chen S. Boyle S. Zhu Y. Zhang A. Piwnica-Worms D. R. (2013). The extracellular domain of Notch2 increases its cell-surface abundance and ligand responsiveness during kidney development. Dev. Cell 25, 585–598. 10.1016/j.devcel.2013.05.022 23806616 PMC3710456

[B89] Liu J. Yang Y. Zheng C. Chen G. Shen Z. Zheng S. (2019). Correlation of interleukin-33/ST2 receptor and liver fibrosis progression in biliary atresia patients. Front. Pediatr. 7, 403. 10.3389/fped.2019.00403 31632941 PMC6781650

[B90] Liu F. Zeng J. Zhu D. Xu X. Lan M. Wang M. (2020). PDGFA gene rs9690350 polymorphism increases biliary atresia risk in Chinese children. Biosci. Rep. 40, BSR20200068. 10.1042/bsr20200068 32662506 PMC7374268

[B91] Liu F. Lui V. C. H. Wu Z. Blakeley P. D. Tang C. S. M. Tam P. K. H. (2024). Animal and organoid models to elucidate the anti-fibrotic effect of steroid on biliary atresia. Pediatr. Surg. Int. 40, 214. 10.1007/s00383-024-05798-7 39102048 PMC11300555

[B92] Lou C. Lan T. Xu S. Hu X. Li J. Xiang Z. (2025). Heterogeneity and plasticity of cholangiocytes in liver injury: a journey from pathophysiology to therapeutic utility. Gut 75, 646–663. 10.1136/gutjnl-2025-334763 40490318 PMC13151499

[B93] Lui V. C. H. (2025). Organoids in biliary atresia. World J. Pediatr. Surg. 8, e001010. 10.1136/wjps-2025-001010 40385243 PMC12083310

[B94] Luo Z. Shivakumar P. Mourya R. Gutta S. Bezerra J. A. (2019). Gene expression signatures associated with survival times of pediatric patients with biliary atresia identify potential therapeutic agents. Gastroenterology 157, 1138–1152.e1114. 10.1053/j.gastro.2019.06.017 31228442 PMC6756963

[B95] Lyu S. Y. Xiao W. Cui G. Z. Yu C. Liu H. Lyu M. (2023). Role and mechanism of DNA methylation and its inhibitors in hepatic fibrosis. Front. Genet. 14, 1124330. 10.3389/fgene.2023.1124330 37056286 PMC10086238

[B96] Lyu H. Ye Y. Wang B. (2023). FIB-4 and APRI scores for progressive liver fibrosis diagnosis in children with biliary atresia. Front. Pediatr. 11, 1286400. 10.3389/fped.2023.1286400 38250586 PMC10796666

[B97] Lyu S. Y. Xiao W. Chen Y. J. Liao Q. L. Cai Y. Y. Yu C. (2025). Multi-parameter magnetic resonance imaging of zebularine in liver fibrosis treatment and calcineurin/NFAT3 mechanism. World J. Gastroenterol. 31, 105554. 10.3748/wjg.v31.i20.105554 40495950 PMC12146936

[B98] Mack C. L. (2007). The pathogenesis of biliary atresia: evidence for a virus-induced autoimmune disease. Semin. Liver Dis. 27, 233–242. 10.1055/s-2007-985068 17682970 PMC3796656

[B99] Mack C. L. Tucker R. M. Sokol R. J. Karrer F. M. Kotzin B. L. Whitington P. F. (2004). Biliary atresia is associated with CD4+ Th1 cell-mediated portal tract inflammation. Pediatr. Res. 56, 79–87. 10.1203/01.Pdr.0000130480.51066.Fb 15128911 PMC1948976

[B100] Mejias M. Gallego J. Naranjo-Suarez S. Ramirez M. Pell N. Manzano A. (2020). CPEB4 increases expression of PFKFB3 to induce glycolysis and activate mouse and human hepatic stellate cells, promoting liver fibrosis. Gastroenterol. 159, 273–288. 10.1053/j.gastro.2020.03.008 32169429

[B101] Meng L. Liu J. Wang J. Du M. Zhang S. Huang Y. (2021). Characteristics of the gut microbiome and IL-13/TGF-β1 mediated fibrosis in post-kasai cholangitis of biliary atresia. Front. Pediatr. 9, 751204. 10.3389/fped.2021.751204 34858903 PMC8630618

[B102] Meng Y. Yang Q. Liu S. Ke X. Zhan J. (2025). Genetic background and biliary atresia. World J. Pediatr. Surg. 8, e001023. 10.1136/wjps-2025-001023 40519535 PMC12161372

[B103] Miller P. N. Baskaran S. Nijagal A. (2024). Immunology of biliary atresia. Semin. Pediatr. Surg. 33, 151474. 10.1016/j.sempedsurg.2025.151474 39862687 PMC12443163

[B104] Mohanty S. K. Lobeck I. Donnelly B. Dupree P. Walther A. Mowery S. (2020). Rotavirus reassortant-induced murine model of liver fibrosis parallels human biliary atresia. Hepatol. 71, 1316–1330. 10.1002/hep.30907 31442322 PMC7384231

[B105] Molina L. Nejak-Bowen K. Monga S. P. (2022). Role of YAP1 signaling in biliary development, repair, and disease. Semin. Liver Dis. 42, 17–33. 10.1055/s-0041-1742277 35073587 PMC9372714

[B106] Nadler E. P. Patterson D. Violette S. Weinreb P. Lewis M. Magid M. S. (2009). Integrin alphavbeta6 and mediators of extracellular matrix deposition are up-regulated in experimental biliary atresia. J. Surg. Res. 154, 21–29. 10.1016/j.jss.2008.05.023 19084240

[B107] Nagi S. A. M. Zakaria H. M. Elkhadry S. Hamed W. E. Gaballa N. K. Elkholy S. S. (2023). APRI and FIB-4 indices as diagnostic noninvasive scores for prediction of severe fibrosis in patients with biliary atresia. Clin. Exp. Hepatol. 9, 251–264. 10.5114/ceh.2023.130699 37790682 PMC10544056

[B108] Naydenov N. G. Ivanov A. I. (2010). Adducins regulate remodeling of apical junctions in human epithelial cells. Mol. Biol. Cell 21, 3506–3517. 10.1091/mbc.E10-03-0259 20810786 PMC2954116

[B109] Nomden M. Beljaars L. Verkade H. J. Hulscher J. B. F. Olinga P. (2020). Current concepts of biliary atresia and matrix metalloproteinase-7: a review of literature. Front. Med. (Lausanne) 7, 617261. 10.3389/fmed.2020.617261 33409288 PMC7779410

[B110] Nusse R. Clevers H. (2017). Wnt/β-Catenin signaling, disease, and emerging therapeutic modalities. Cell 169, 985–999. 10.1016/j.cell.2017.05.016 28575679

[B111] Nyholm I. Hukkinen M. Pakarinen M. P. (2024). Predicting and managing liver fibrosis in biliary atresia. Semin. Pediatr. Surg. 33, 151473. 10.1016/j.sempedsurg.2025.151473 39884181

[B112] Omenetti A. Diehl A. M. (2011). Hedgehog signaling in cholangiocytes. Curr. Opin. Gastroenterol. 27, 268–275. 10.1097/MOG.0b013e32834550b4 21423008 PMC3636549

[B113] Omenetti A. Bass L. M. Anders R. A. Clemente M. G. Francis H. Guy C. D. (2011a). Hedgehog activity, epithelial-mesenchymal transitions, and biliary dysmorphogenesis in biliary atresia. Hepatol. 53, 1246–1258. 10.1002/hep.24156 21480329 PMC3074103

[B114] Omenetti A. Choi S. Michelotti G. Diehl A. M. (2011b). Hedgehog signaling in the liver. J. Hepatol. 54, 366–373. 10.1016/j.jhep.2010.10.003 21093090 PMC3053023

[B115] Ortiz-Perez A. Donnelly B. Temple H. Tiao G. Bansal R. Mohanty S. K. (2020). Innate immunity and pathogenesis of biliary atresia. Front. Immunol. 11, 329. 10.3389/fimmu.2020.00329 32161597 PMC7052372

[B193] Ozturk A. Olson M. C. Samir A. E. Venkatesh S. K. (2022). Liver fibrosis assessment: MR and US elastography. Abdom. Radiol. (NY) 47, 3037–3050. 10.1007/s00261-021-03269-4 34687329 PMC9033887

[B116] Pan D. Zheng T. Chen C. Qiu J. Deng Z. Jiang Z. (2026). Single-cell transcriptomics reveals the ameliorative effect of gastrodin on cholestatic liver fibrosis. Phytomedicine 155, 158165. 10.1016/j.phymed.2026.158165 41999704

[B117] Pandurangi S. Mourya R. Nalluri S. Fei L. Dong S. Harpavat S. (2024). Diagnostic accuracy of serum matrix metalloproteinase-7 as a biomarker of biliary atresia in a large North American cohort. Hepatol. 80, 152–162. 10.1097/hep.0000000000000827 PMC11191042 38446707

[B118] Park S. M. (2012). The crucial role of cholangiocytes in cholangiopathies. Gut Liver 6, 295–304. 10.5009/gnl.2012.6.3.295 22844556 PMC3404165

[B119] Peng L. Hu Y. Zhang X. Tan C. Yang C. Liu T. (2025). TGF-β1/SMAD3-mediated non-canonical hedgehog signaling promotes pancreatic stellate cell activation and fibrosis in chronic pancreatitis. Int. J. Biol. Sci. 21, 6978–6996. 10.7150/ijbs.108149 41281756 PMC12631176

[B120] Perakakis N. Stefanakis K. Mantzoros C. S. (2020). The role of omics in the pathophysiology, diagnosis and treatment of non-alcoholic fatty liver disease. Metabolism 111s, 154320. 10.1016/j.metabol.2020.154320 32712221 PMC7377759

[B121] Priester S. Wise C. Glaser S. S. (2010). Involvement of cholangiocyte proliferation in biliary fibrosis. World J. Gastrointest. Pathophysiol. 1, 30–37. 10.4291/wjgp.v1.i2.30 21607140 PMC3097945

[B122] Qiu J. L. Zhang G. F. Chai Y. N. Han X. Y. Zheng H. T. Li X. F. (2022). Ligustrazine attenuates liver fibrosis by targeting miR-145 mediated transforming growth factor-β/smad signaling in an animal model of biliary atresia. J. Pharmacol. Exp. Ther. 381, 257–265. 10.1124/jpet.121.001020 35398813

[B123] Rajagopalan R. Tsai E. A. Grochowski C. M. Kelly S. M. Loomes K. M. Spinner N. B. (2020). Exome sequencing in individuals with isolated biliary atresia. Sci. Rep. 10, 2709. 10.1038/s41598-020-59379-4 32066793 PMC7026070

[B124] Ramm G. A. Nair V. G. Bridle K. R. Shepherd R. W. Crawford D. H. (1998). Contribution of hepatic parenchymal and nonparenchymal cells to hepatic fibrogenesis in biliary atresia. Am. J. Pathol. 153, 527–535. 10.1016/s0002-9440(10)65595-2 9708812 PMC1852970

[B125] Rigamonti A. E. Bondesan A. Rondinelli E. Cella S. G. Sartorio A. (2022). The role of aspartate transaminase to platelet ratio index (APRI) for the prediction of non-alcoholic fatty liver disease (NAFLD) in severely obese children and adolescents. Metabolites 12, 155. 10.3390/metabo12020155 35208229 PMC8879448

[B126] Russi A. E. Shivakumar P. Luo Z. Bezerra J. A. (2023). Plasticity between type 2 innate lymphoid cell subsets and amphiregulin expression regulates epithelial repair in biliary atresia. Hepatol. 78, 1035–1049. 10.1097/hep.0000000000000418 PMC10524120 37078450

[B127] Sanada Y. Sakuma Y. Onishi Y. Okada N. Hirata Y. Horiuchi T. (2022). Long-term outcomes in pediatric patients who underwent living donor liver transplantation for biliary atresia. Surgery 171, 1671–1676. 10.1016/j.surg.2021.11.027 35027207

[B128] Sandberg J. K. Sun Y. Ju Z. Liu S. Jiang J. Koci M. (2021). Ultrasound shear wave elastography: does it add value to gray-scale ultrasound imaging in differentiating biliary atresia from other causes of neonatal jaundice? Pediatr. Radiol. 51, 1654–1666. 10.1007/s00247-021-05024-9 33772640

[B129] Schmidt H. C. Hagens J. Schuppert P. Appl B. Raluy L. P. Trochimiuk M. (2023). Biliatresone induces cholangiopathy in C57BL/6J neonates. Sci. Rep. 13, 10574. 10.1038/s41598-023-37354-z 37386088 PMC10310722

[B130] Schön P. Tsuchiya K. Lenoir D. Mochizuki T. Guichard C. Takai S. (2002). Identification, genomic organization, chromosomal mapping and mutation analysis of the human INV gene, the ortholog of a murine gene implicated in left-right axis development and biliary atresia. Hum. Genet. 110, 157–165. 10.1007/s00439-001-0655-5 11935322

[B131] Schuppan D. (2015). Liver fibrosis: common mechanisms and antifibrotic therapies. Clin. Res. Hepatol. Gastroenterol. 39, S51–S59. 10.1016/j.clinre.2015.05.005 26189980

[B132] Shamsan E. Almezgagi M. Gamah M. Khan N. Qasem A. Chuanchuan L. (2024). The role of PI3k/AKT signaling pathway in attenuating liver fibrosis: a comprehensive review. Front. Med. (Lausanne) 11, 1389329. 10.3389/fmed.2024.1389329 38590313 PMC10999701

[B133] Shaalan U. F. Ibrahim N. L. Ehsan N. A. Sultan M. M. Naser G. M. Abd El-Fatah M. O. (2019). Reduced immunohistochemical expression of Hnf1β and FoxA2 in liver tissue can discriminate between biliary atresia and other causes of neonatal cholestasis. Appl. Immunohistochem. Mol. Morphol. 27, e32–e38. 10.1097/pai.0000000000000638 29406331

[B134] Shan L. Wang F. Zhai D. Meng X. Liu J. Lv X. (2024). Kasai portoenterostomy, successful liver transplantation, and immunosuppressive therapy for biliary atresia in a female baby: a case report. J. Inflamm. Res. 17, 4905–4920. 10.2147/jir.S432024 39070130 PMC11283245

[B135] Sharip A. Kunz J. (2025). Mechanosignaling via integrins: pivotal players in liver fibrosis progression and therapy. Cells 14, 266. 10.3390/cells14040266 39996739 PMC11854242

[B136] Shashni B. Tajika Y. Ikeda Y. Nishikawa Y. Nagasaki Y. (2023). Self-assembling polymer-based short chain fatty acid prodrugs ameliorate non-alcoholic steatohepatitis and liver fibrosis. Biomaterials 295, 122047. 10.1016/j.biomaterials.2023.122047 36840994

[B137] Shen W. J. Dong R. Chen G. Zheng S. (2014). microRNA-222 modulates liver fibrosis in a murine model of biliary atresia. Biochem. Biophys. Res. Commun. 446, 155–159. 10.1016/j.bbrc.2014.02.065 24569080

[B138] Shen W. J. Chen G. Wang M. Zheng S. (2019). Liver fibrosis in biliary atresia. World J. Pediatr. 15 (2), 117–123. 10.1007/s12519-018-0203-1 30465125

[B139] Siyu P. Junxiang W. Qi W. Yimao Z. Shuguang J. (2022). The role of GLI in the regulation of hepatic epithelial-mesenchymal transition in biliary atresia. Front. Pediatr. 10, 861826. 10.3389/fped.2022.861826 35692978 PMC9178093

[B140] So J. Ningappa M. Glessner J. Min J. Ashokkumar C. Ranganathan S. (2020). Biliary atresia-associated mannosidase-1-alpha-2 gene regulates biliary and ciliary morphogenesis and laterality. Front. Physiol. 11, 538701. 10.3389/fphys.2020.538701 33192543 PMC7662016

[B141] Squires J. H. Fetzer D. T. Dillman J. R. (2022). Practical contrast enhanced liver ultrasound. Radiol. Clin. North Am. 60, 717–730. 10.1016/j.rcl.2022.04.006 35989040

[B142] Sumida Y. Yoneda M. Tokushige K. Kawanaka M. Fujii H. Yoneda M. (2021). FIB-4 first in the diagnostic algorithm of metabolic-dysfunction-associated fatty liver disease in the era of the global metabodemic. Life (Basel) 11, 143. 10.3390/life11020143 33672864 PMC7917687

[B143] Sutton H. Karpen S. J. Kamath B. M. (2024). Pediatric cholestatic diseases: common and unique pathogenic mechanisms. Annu. Rev. Pathol. 19, 319–344. 10.1146/annurev-pathmechdis-031521-025623 38265882

[B144] Tam P. K. H. Wells R. G. Tang C. S. M. Lui V. C. H. Hukkinen M. Luque C. D. (2024). Biliary atresia. Nat. Rev. Dis. Prim. 10, 47. 10.1038/s41572-024-00533-x 38992031 PMC11956545

[B145] Theodoropoulou M. A. Mantzourani C. Kokotos G. (2024). Histone Deacetylase (HDAC) inhibitors as a novel therapeutic option against fibrotic and inflammatory diseases. Biomolecules 14, 1605. 10.3390/biom14121605 39766311 PMC11674560

[B146] Tian L. Wang Y. Jang Y. Y. (2022). Wnt signaling in biliary development, proliferation, and fibrosis. Exp. Biol. Med. (Maywood) 247, 360–367. 10.1177/15353702211061376 34861115 PMC8899336

[B147] Tran K. T. Le V. S. Dao L. T. M. Nguyen H. K. Mai A. K. Nguyen H. T. (2021). Novel findings from family-based exome sequencing for children with biliary atresia. Sci. Rep. 11, 21815. 10.1038/s41598-021-01148-y 34750413 PMC8575792

[B148] Trivedi P. Wang S. Friedman S. L. (2021). The power of plasticity-metabolic regulation of hepatic stellate cells. Cell Metab. 33, 242–257. 10.1016/j.cmet.2020.10.026 33232666 PMC7858232

[B149] Udomsinprasert W. Ungsudechachai T. Vejchapipat P. Poovorawan Y. Honsawek S. (2022). Systemic cytokine profiles in biliary atresia. PLoS One 17, e0267363. 10.1371/journal.pone.0267363 35452452 PMC9032369

[B150] Van Tung N. Lien N. T. K. Lan N. N. Mai N. T. P. Yen P. T. H. Hoa N. P. A. (2021). The role of p.Val444Ala variant in the ABCB11 gene and susceptibility to biliary atresia in Vietnamese patients. Med. (Baltimore) 100, e28011. 10.1097/md.0000000000028011 PMC8615439 34964797

[B151] Vij M. Rela M. (2020). Biliary atresia: pathology, etiology and pathogenesis. Future Sci. OA 6, Fso466. 10.2144/fsoa-2019-0153 32518681 PMC7273417

[B152] Wang Y. Shen R. W. Han B. Li Z. Xiong L. Zhang F. Y. (2017). Notch signaling mediated by TGF-β/Smad pathway in concanavalin A-induced liver fibrosis in rats. World J. Gastroenterol. 23, 2330–2336. 10.3748/wjg.v23.i13.2330 28428712 PMC5385399

[B153] Wang J. Y. Cheng H. Zhang H. Y. Ye Y. Q. Feng Q. Chen Z. M. (2019). Suppressing microRNA-29c promotes biliary atresia-related fibrosis by targeting DNMT3A and DNMT3B. Cell Mol. Biol. Lett. 24, 10. 10.1186/s11658-018-0134-9 30906331 PMC6410490

[B154] Wang J. Xu Y. Chen Z. Liang J. Lin Z. Liang H. (2020). Liver immune profiling reveals pathogenesis and therapeutics for biliary atresia. Cell 183, 1867–1883.e1826. 10.1016/j.cell.2020.10.048 33248023

[B155] Wang H. Geng C. Zhou H. Zhang Z. Chen W. (2021). Cyclopamine sensitizes multiple myeloma cells to circularly permuted TRAIL-induced apoptosis. Oncol. Lett. 21, 295. 10.3892/ol.2021.12556 33732371 PMC7905534

[B156] Wang X. Zhang R. Lin Z. Fu M. Chen Y. (2023). A mouse model of chronic liver fibrosis for the study of biliary atresia. J. Vis. Exp., 192. 10.3791/65044 36807296

[B157] Wang J. Du M. Meng L. Yang Y. He S. Zhu Y. (2023). Integrative analysis implicates the significance of m6A in the liver fibrosis of biliary atresia by regulating THY1. Hepatol. Commun. 7 (1), e0004. 10.1097/HC9.0000000000000004 36633486 PMC9827977

[B158] Wang F. Chen L. Kong D. Zhang X. Xia S. Liang B. (2024). Canonical Wnt signaling promotes HSC glycolysis and liver fibrosis through an LDH-A/HIF-1α transcriptional complex. Hepatol. 79, 606–623. 10.1097/hep.0000000000000569 PMC10871634 37733267

[B159] Wang C. X. Hou J. J. Lin S. Y. Wang J. H. Ding J. J. Liu C. (2025). Association between liver fibrosis’s noninvasive scores and retinal imaging changes: insights from NHANES. J. Health Popul. Nutr. 44, 56. 10.1186/s41043-025-00805-6 40022221 PMC11871793

[B160] Wang H. Liu H. Li W. Keranmu A. Liang P. Wang Y. (2025). Effect of serum MMP-7 on the diagnostic accuracy of biliary atresia: systematic review and meta-analysis. Front. Pharmacol. 16, 1581053. 10.3389/fphar.2025.1581053 40642008 PMC12240794

[B161] Wells R. G. (2008). Cellular sources of extracellular matrix in hepatic fibrosis. Clin. Liver Dis. 12, 759–768. 10.1016/j.cld.2008.07.008 18984465 PMC2617723

[B162] Wilson C. L. Mann D. A. Borthwick L. A. (2017). Epigenetic reprogramming in liver fibrosis and cancer. Adv. Drug Deliv. Rev. 121, 124–132. 10.1016/j.addr.2017.10.011 29079534 PMC5716427

[B163] Wu L. N. Zhu Z. J. Sun L. Y. (2022). Genetic factors and their role in the pathogenesis of biliary atresia. Front. Pediatr. 10, 912154. 10.3389/fped.2022.912154 35844731 PMC9277099

[B164] Xiao Y. Zhou Y. Chen Y. Zhou K. Wen J. Wang Y. (2015). The expression of epithelial-mesenchymal transition-related proteins in biliary epithelial cells is associated with liver fibrosis in biliary atresia. Pediatr. Res. 77, 310–315. 10.1038/pr.2014.181 25406900

[B165] Xiao Y. Liu R. Li X. Gurley E. C. Hylemon P. B. Lu Y. (2019). Long noncoding RNA H19 contributes to cholangiocyte proliferation and cholestatic liver fibrosis in biliary atresia. Hepatol. 70 (5), 1658–1673. 10.1002/hep.30698 31063660 PMC6819224

[B166] Xiao M. H. Wu S. Liang P. Ma D. Zhang J. Chen H. (2024). Mucosal-associated invariant T cells promote ductular reaction through amphiregulin in biliary atresia. EBioMedicine 103, 105138. 10.1016/j.ebiom.2024.105138 38678809 PMC11077624

[B167] Xie H. Zhu Z. Tang J. Zhu W. Zhu M. Yi Wai A. W. (2025). Dysregulated activation of Hippo-YAP1 signaling induces oxidative stress and aberrant development of intrahepatic biliary cells in biliary atresia. Lab. Invest. 105, 102199. 10.1016/j.labinv.2024.102199 39579985

[B168] Xing G. D. Wang X. Q. Duan L. Liu G. Wang Z. Xiao Y. H. (2024). Robotic-assisted Kasai portoenterostomy for child biliary atresia. World J. Gastrointest. Surg. 16, 3780–3785. 10.4240/wjgs.v16.i12.3780 39734449 PMC11650233

[B169] Xu G. Ma T. Zhou C. Zhao F. Peng K. Li B. (2024). Combination of Pirfenidone and andrographolide ameliorates hepatic stellate cell activation and liver fibrosis by mediating TGF-β/Smad signaling pathway. Anal. Cell Pathol. (Amst) 2024, 2751280. 10.1155/2024/2751280 38946862 PMC11213636

[B170] Xue Y. Zhu W. Qiao F. Yang Y. Qiu J. Zou C. (2024). Ba-Qi-Rougan formula alleviates hepatic fibrosis by suppressing hepatic stellate cell activation via the MSMP/CCR2/PI3K pathway. J. Ethnopharmacol. 329, 118169. 10.1016/j.jep.2024.118169 38621463

[B171] Yang Y. Jin Z. Dong R. Zheng C. Huang Y. Zheng Y. (2018). MicroRNA-29b/142-5p contribute to the pathogenesis of biliary atresia by regulating the IFN-γ gene. Cell Death Dis. 9, 545. 10.1038/s41419-018-0605-y 29748604 PMC5945737

[B172] Yang Y. Ni M. Zong R. Yu M. Sun Y. Li J. (2023). Targeting Notch1-YAP circuit reprograms macrophage polarization and alleviates acute liver injury in mice. Cell Mol. Gastroenterol. Hepatol. 15, 1085–1104. 10.1016/j.jcmgh.2023.01.002 36706917 PMC10036742

[B173] Yang T. Yang S. Mou W. Zhao J. Wang H. Chen Y. (2025). SULT2B1 promotes cholangiocyte epithelial-mesenchymal transition via Wnt/β-catenin/MMP7 pathway in biliary atresia. Pediatr. Res. 10.1038/s41390-025-04304-6 PMC13485696 41402642

[B174] Ye C. Zhu J. Wang J. Chen D. Meng L. Zhan Y. (2022). Single-cell and spatial transcriptomics reveal the fibrosis-related immune landscape of biliary atresia. Clin. Transl. Med. 12 (11), e1070. 10.1002/ctm2.1070 36333281 PMC9636046

[B175] Ying X. Dong S. Zhao Y. Chen Z. Jiang J. Shi H. (2025). Research progress on contrast-enhanced ultrasound (CEUS) assisted diagnosis and treatment in liver-related diseases. Int. J. Med. Sci. 22, 1092–1108. 10.7150/ijms.101789 40027182 PMC11866529

[B176] Yoon H. Ihn K. Kim J. Lim H. J. Park S. Han S. J. (2023). Pre-and immediate post-Kasai portoenterostomy shear wave elastography for predicting hepatic fibrosis and native liver outcomes in patients with biliary atresia. Korean J. Radiol. 24, 465–475. 10.3348/kjr.2022.0586 37056157 PMC10157319

[B177] Zagory J. A. Nguyen M. V. Wang K. S. (2015). Recent advances in the pathogenesis and management of biliary atresia. Curr. Opin. Pediatr. 27, 389–394. 10.1097/mop.0000000000000214 25944310 PMC4447429

[B178] Zeng S. Sun P. Chen Z. Mao J. Wang J. Wang B. (2014). Association between single nucleotide polymorphisms in the ADD3 gene and susceptibility to biliary atresia. PLoS One 9, e107977. 10.1371/journal.pone.0107977 25285724 PMC4186752

[B179] Zeng J. Fan J. Zhou H. (2023). Bile acid-mediated signaling in cholestatic liver diseases. Cell Biosci. 13, 77. 10.1186/s13578-023-01035-1 37120573 PMC10149012

[B180] Zeng X. Liao Y. Qiao X. Liang K. Luo Q. Deng M. (2023). Novel NIR-II fluorescent probes for biliary atresia imaging. Acta Pharm. Sin. B 13, 4578–4590. 10.1016/j.apsb.2023.07.005 37969732 PMC10638547

[B181] Zeybel M. Hardy T. Wong Y. K. Mathers J. C. Fox C. R. Gackowska A. (2012). Multigenerational epigenetic adaptation of the hepatic wound-healing response. Nat. Med. 18, 1369–1377. 10.1038/nm.2893 22941276 PMC3489975

[B182] Zhan J. Liu S. Meng Y. Yang Q. Wang Z. Zhang S. (2024a). Systematic review of the mechanism and assessment of liver fibrosis in biliary atresia. Pediatr. Surg. Int. 40, 205. 10.1007/s00383-024-05778-x 39033225

[B183] Zhan J. Liu S. Li T. Li X. (2024b). Kasai procedure or liver transplantation: how should we choose in biliary atresia? Hepatobiliary Surg. Nutr. 13, 1019–1021. 10.21037/hbsn-24-509 39669068 PMC11634429

[B184] Zhang H. Zhang M. (2025). Perioperative management of a neonate with congenital biliary atresia complicated by severe pneumonia undergoing hepatic hilar jejunostomy: a case report. Nurs. Crit. Care 30 (5), e13263. 10.1111/nicc.13263 39887839

[B185] Zhang R. Z. Yu J. K. Peng J. Wang F. H. Liu H. Y. Lui V. C. H. (2016). Role of CD56-expressing immature biliary epithelial cells in biliary atresia. World J. Gastroenterol. 22, 2545–2557. 10.3748/wjg.v22.i8.2545 26937142 PMC4768200

[B186] Zheng D. Jiang Y. Qu C. Yuan H. Hu K. He L. (2020). Pyruvate kinase M2 tetramerization protects against hepatic stellate cell activation and liver fibrosis. Am. J. Pathol. 190, 2267–2281. 10.1016/j.ajpath.2020.08.002 32805235 PMC7786052

[B187] Zhou W. Zhou L. (2021). Ultrasound for the diagnosis of biliary atresia: from conventional ultrasound to artificial intelligence. Diagn. (Basel) 12, 51. 10.3390/diagnostics12010051 PMC8775261 35054217

[B192] Zhou W. Liang J. Shan Q. Chen H. Gao P. Cao Q. (2022). Comparison of two kinds of two-dimensional shear wave elastography techniques in the evaluation of jaundiced infants suspected of biliary atresia. Diagnostics (Basel). 12 (5), 1092. 10.3390/diagnostics12051092 35626253 PMC9140168

[B188] Zhou Y. Ji H. Xu Q. Zhang X. Cao X. Chen Y. (2021). Congenital biliary atresia is correlated with disrupted cell junctions and polarity caused by Cdc42 insufficiency in the liver. Theranostics 11, 7262–7275. 10.7150/thno.49116 34158849 PMC8210598

[B189] Zhu J. J. Yang Y. F. Dong R. Zheng S. (2023). Biliatresone: progress in biliary atresia study. World J. Pediatr. 19, 417–424. 10.1007/s12519-022-00619-0 36166189 PMC10149470

[B190] Zhu Z. Lu C. Lv X. Lu Y. Zhu W. Zhu Y. (2026). EP300/YAP1-SERPINE1 signaling regulates ductular reaction and liver fibrosis in biliary atresia. Cell Mol. Gastroenterol. Hepatol. 20, 101640. 10.1016/j.jcmgh.2025.101640 40992734 PMC12630365

[B191] Zhubanchaliyev A. Temirbekuly A. Kongrtay K. Wanshura L. C. Kunz J. (2016). Targeting mechanotransduction at the transcriptional level: YAP and BRD4 are novel therapeutic targets for the reversal of liver fibrosis. Front. Pharmacol. 7, 462. 10.3389/fphar.2016.00462 27990121 PMC5131002

